# Amino acid availability influences *Staphylococcus aureus* interaction with the nasal microbiota in a strain- and community-specific manner

**DOI:** 10.1186/s40168-026-02563-x

**Published:** 2026-10-23

**Authors:** Laura Camus, Jessica Franz, David Gerlach, Anna Lange, Jeffrey John Power, Marcelo Navarro-Díaz, Jens Stahl, Bernhard Krismer, Johanna Rapp, Kevser Bilici, Matthias Otto, Levin Joe Klages, Vladimir Starostin, Soyoung Ham, Jörn Kalinowski, Largus Theodora Angenent, Hannes Link, Simon Heilbronner

**Affiliations:** 1 Interfaculty Institute of Microbiology and Infection Medicine, Department of Infection Biology, University of Tübingen, 72076 Tübingen, Germany; 2 Cluster of Excellence EXC 2124 Controlling Microbes to Fight Infections, 72076 Tübingen, Germany; 3 German Centre for Infection Research (DZIF), Partner Site Tübingen, Tübingen, Germany; 4 Faculty of Biology, Microbiology, Ludwig-Maximilians-University of Munich, 82152 Munich, Germany; 5 M3 Research Center, University of Tübingen, Otfried-Müller-Straße 37, 72076 Tübingen, Germany; 6 Center for Biotechnology (CeBiTec), Bielefeld University, 33615 Bielefeld, Germany; 7 Cluster of Excellence Machine Learning: New Perspectives for Science, University of Tübingen, Maria-Von-Linden-Straße 6, 72076 Tübingen, Germany; 8 Environmental Biotechnology Group, Department of Geosciences, University of Tübingen, 72076 Tübingen, Germany

**Keywords:** *Staphylococcus aureus*, Nasal colonization, Commensal microbiota, Synthetic communities, Bacterial interactions, Amino acid auxotrophy, Tyrosine metabolism, Gnotobiotic mice

## Abstract

**Background:**

Only some individuals carry *Staphylococcus aureus* in the nose, predisposing them to endogenous infections. While microbial cooperation and competition are known to shape environmental and intestinal microbial communities, their impact on pathogen colonization within the nasal microbiome is largely unknown.

**Results:**

Using a newly curated nasal strain collection and systematic pairwise interaction analysis, we investigated competitive and collaborative traits between diverse *S. aureus* strains and nasal communities. Our experiments revealed that nasal species strongly modulate *S. aureus* growth in a strain- and community-specific manner both in vitro and in gnotobiotic mice. Nutrient-dependent interactions emerged as key drivers. In one example, we found that tyrosine provided by synthetic communities supported proliferation of an auxotrophic *S. aureus* strain. Screening of a large collection of *S. aureus* strains identified tyrosine dependency in 8% of strains, with colonization impaired in the absence of supportive communities, indicating broader relevance of this interaction.

**Conclusions:**

Our findings reveal distinct metabolic adaptations that enable *S. aureus* to exploit specific features of resident nasal communities, highlighting complex interspecies networks that influence colonization in the human nose. These insights may inform microbiome-editing strategies to displace *S. aureus* from the nasal niche.

Video Abstract

**Graphical Abstract:**

**Figure:**
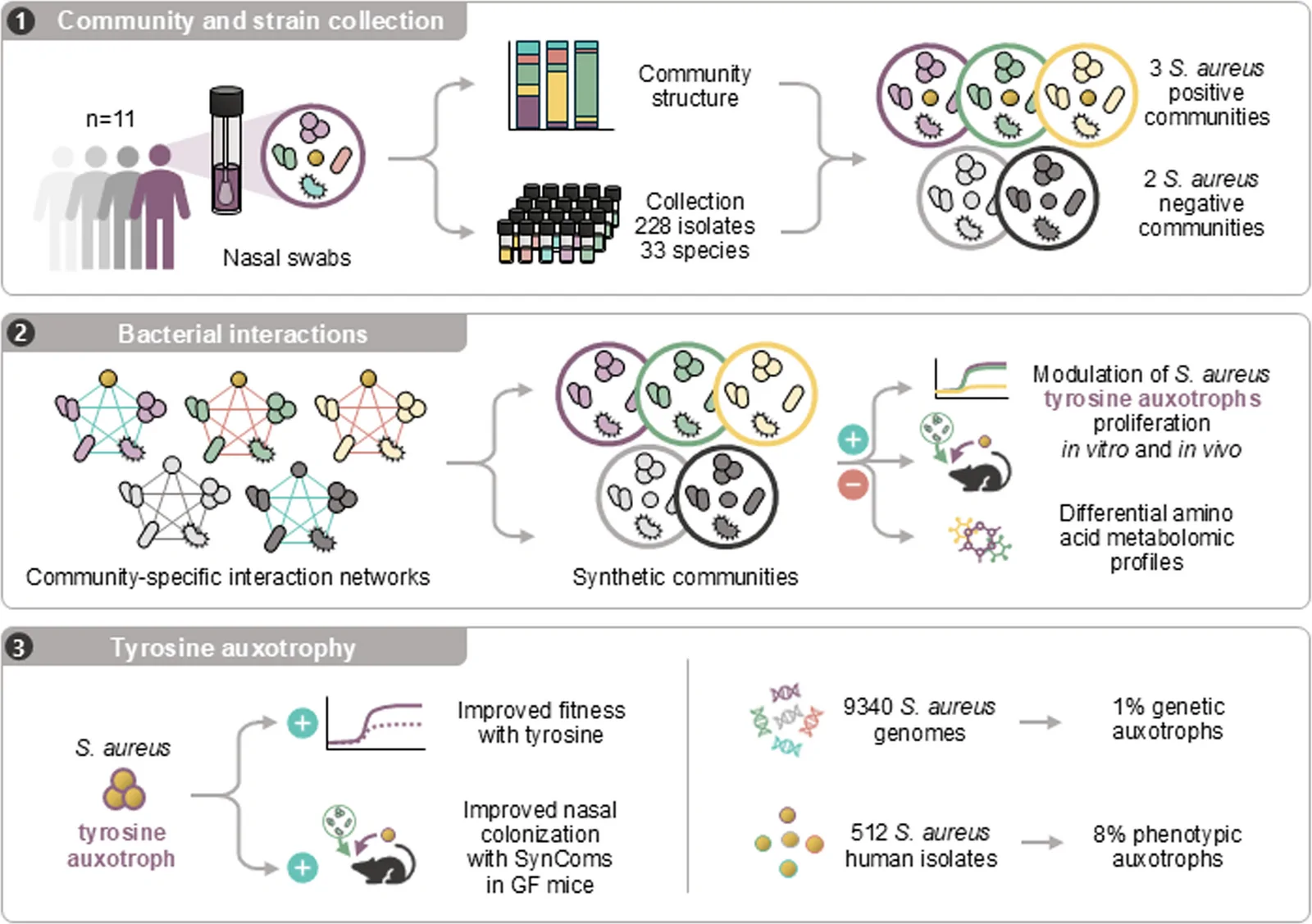

**Supplementary Information:**

The online version contains supplementary material available at https://doi.org/10.1186/s40168-026-02563-x.

## Introduction

Host-associated microbial communities often act as reservoirs for opportunistic pathogens, thereby increasing the risk of infection. However, it has been recognized that certain resident species can provide colonization resistance to invader species. Besides production of antibacterial and quorum-quenching molecules, nutritional competition has emerged as a crucial defense mechanism of the microbiota, particularly within the nutrient-rich surrounding of the human intestinal tract [1–3]. The nasal ecosystem can also harbor pathogens, with *Staphylococcus aureus* constituting the greatest threat for human health due to its ability to cause a broad spectrum of infections [4]. Within the human population, individuals may be either permanently, transiently, or never colonized by *S. aureus,* although the existence of the transient carriage is debated [5–8]*.* The molecular reasons underlying colonization status remain largely unclear. While carriage is considered a non-heritable trait, it has been shown to be impacted by host immune status [4, 8], as well as by a range of other host and environmental factors, including sex, age, hygiene practices, and level of exposure to *S. aureus* [7–11]. On the bacterial side, a number of *S. aureus* factors have been identified as contributing to nasal colonization. These include components involved in adherence to host cells, such as adhesins or wall teichoic acids, as well as mechanisms conferring resistance to antibacterial fatty acids [4, 8].

The resident nasal microbiota also appears to influence *S. aureus* colonization, and several studies explored community signatures associated with *S. aureus* carriage through culture-dependent or -independent approaches. Using 16S rRNA gene sequencing, Liu et al. defined seven nasal community state types (CSTs) based on the dominant taxa [9], which were recently expanded to nine CSTs (CST1–9) [10] or reclassified into alternative groups [7]. Despite differences likely arising from cohort composition and analytical approaches, both metagenomic and culture-based studies converge to identifying *S. aureus* as a central member of the nasal ecosystem. This is supported by (i) its high prevalence and abundance across cohorts, (ii) its association with specific community structures (CST1/I), and (iii) its high degree of centrality and connections to other species, as identified through co-occurrence analyses [7, 10, 12]. More importantly, these studies evidence recurrent negative and positive associations between *S. aureus* and other nasal taxa. For instance, *S. aureus* carriage has been negatively associated with the presence of *Dolosigranulum pigrum *[9, 10, 13, 14], *S. epidermidis *[15–21], and *S. lugdunensis *[22, 23]. The three species were also shown to inhibit *S. aureus *in vitro, notably through the use of secreted factors such as bacteriocins [14, 17, 20, 22, 24]. Negative correlations have also been reported with *Finegoldia magna* and *Corynebacterium* spp (particularly *C. propinquum* and *C. pseudodiphtheriticum*) [7, 10, 13, 18, 25, 26]. The molecular basis of these patterns remains to be characterized but is thought to involve secreted factors and/or metabolic competition [27, 28]. In contrast, *Corynebacterium accolens* and *Cutibacterium acnes,* two common nasal colonizers, have been reported to exhibit both negative [7, 10, 19] and positive [13, 26, 29] relationships with *S. aureus* abundance and/or in vitro behavior.

However, emerging evidence also suggests that observed interactions are often strain- rather than species-specific, and can be caused by the production of antimicrobial compounds, iron-scavenging molecules, and other competitive factors by certain nasal isolates [21, 30]. Strain-specific interactions, along with co-evolutionary dynamics between co-colonizing strains, have been shown to play a critical role in determining the structure, function, and stability of microbial communities in various ecosystems, including the plant microbiome [31] and the human gut [32, 33]. Despite this well-established role in other microbial ecosystems, it remains largely unexplored whether similar strain-specific traits and co-adaptation mechanisms shape the interactions of *S. aureus* with nasal communities. Moreover, interactions between *S. aureus* and nasal species have typically been investigated in a pairwise fashion. Yet, the outcome of such interactions in natural, complex communities remains uncertain as they can be influenced by higher-order interactions [34].

In complex microbial communities, resource availability and its distribution among members are key factors shaping community structure and resistance to invasion by other species [1, 2, 35]. The nasal environment is characterized by low concentrations of both nutrients and bacteria [9, 36], a context likely to favor supportive behaviors among microorganisms to optimize resource utilization [35]. The Synthetic Nasal Medium (SNM) has been developed to recapitulate key nutritional features of the nasal environment in vitro [36]*.* Nasal secretions and, consequently, SNM, are characterized by low content of the carbon and energy sources glucose, pyruvate and succinate. Additionally, amino acids are present in low concentrations, with six of them (methionine, glutamine, tyrosine, isoleucine, asparagine, and aspartate) completely absent. Among these, methionine limitation has been shown to be relevant for *S. aureus* nasal colonization [36]. Despite this knowledge and experimental options, our understanding of *S. aureus* interactions with nasal species within the nutritional environment of the human nasal cavity remains limited. The structure of nutritional networks and their impact on pathogen proliferation have yet to be elucidated. Furthermore, it remains unknown how strain-dependent heterogeneity in *S. aureus* influences its interactions with the nasal microbiota.

In this study, we established a nasal strain collection that captures intra-volunteer diversity at both the species- and strain-level in healthy adults. Using this collection, we investigated the interactions between *S. aureus* and nasal species through both pairwise assays and within synthetic consortia in vitro and within gnotobiotic animals. In nutrient-poor conditions mimicked by SNM, we found that these interactions are predominantly supportive and shaped by strain- and community-specific factors. Notably, we found that tyrosine supplementation stimulated the growth of 8.2% of nasal *S. aureus* strains in SNM, suggesting that these strains may rely on amino acid-releasing microbial communities to proliferate in the nutrient-poor environment of the nasal cavity.

## Results

### Establishment of the nasal strain collection LaCa

**Fig. 1:**
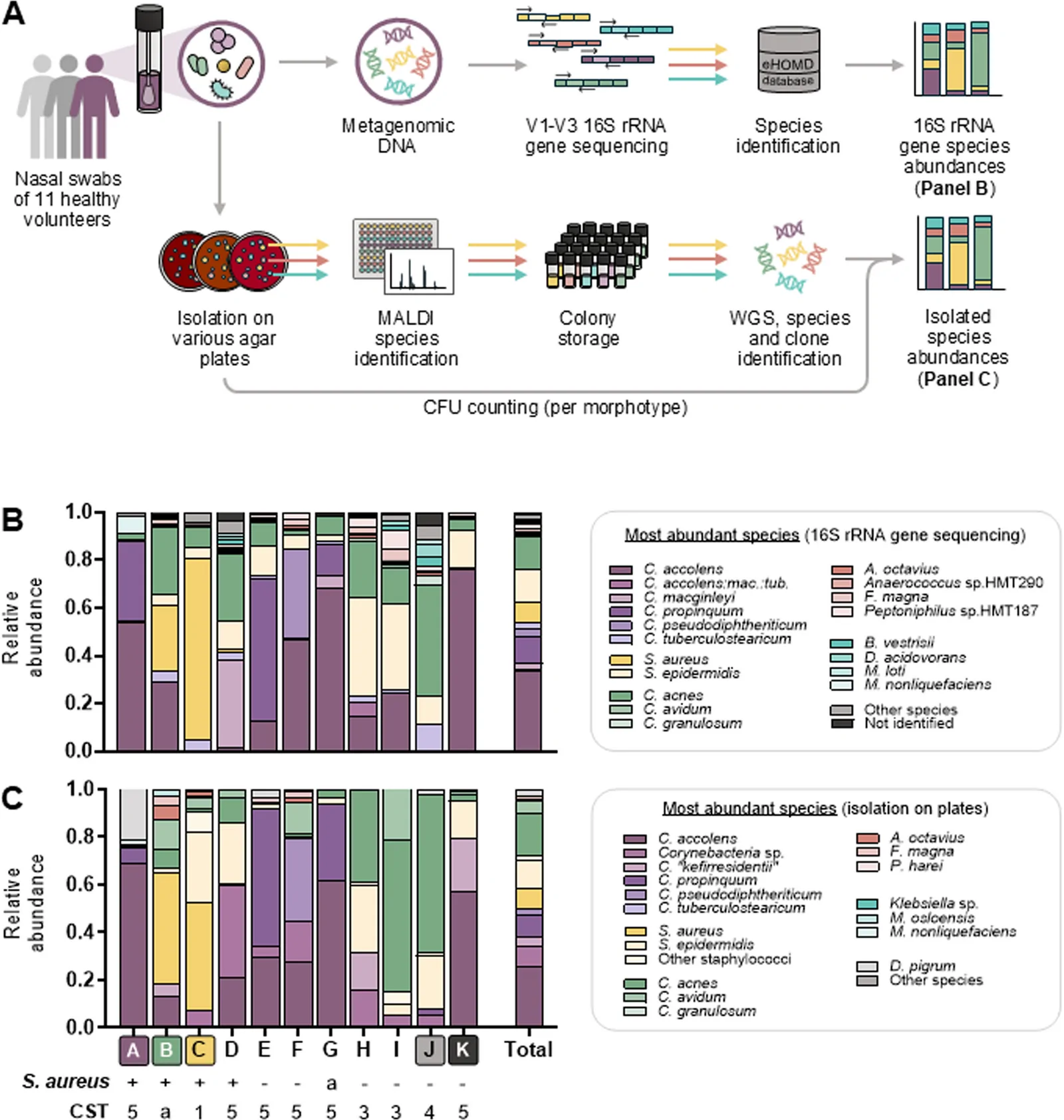
Species composition and structure of 11 nasal communities.** A** Schematic methodology used to determine nasal community structures and establish the strain collection LaCa. **B, C** Relative species abundance determined by 16S rRNA gene sequencing of nasal metagenomic DNA (**B**) or by isolation and enumeration of nasal species on plates (**C**), from the nasal microbiomes of eleven healthy volunteers (**A**–**K**). Each color designates one of the 20 most abundant species across all volunteers. Less abundant species were assigned to “Other species”. Species with a missing or unclear identification were assigned to “Not identified”. Below the bars, “ + ” indicates the volunteers in which *S. aureus* was detected by both techniques, and numbers correspond to the community state type (CST) assigned to the corresponding community. “a” refers to ambiguous *S. aureus* carriage (detected by only one method) or CST classification (balanced proportion of several species). Each bar displays the results of one replicate. See also Supplementary Fig. S1 and Tables S1, S2, and S3

We established a nasal biobank to reconstruct nasal microbial communities at the strain level. A cohort of eleven healthy volunteers was recruited as detailed in the Methods section. The structure of their nasal microbiome was determined using 16S rRNA gene amplicon sequencing, and bacterial strains were isolated from their anterior nares (Fig. 1A). The analysis of the 16S rRNA gene amplicon sequencing showed the species *C. accolens, C. acnes, S. epidermidis, C. propinquum* and *S. aureus* to account for 80% of the total abundance across all volunteers, largely reflecting the nasal core species identified in previous studies [9, 19, 26]. The species *C. acnes, S. epidermidis, C. tuberculostearicum* and *C. granulosum* were detected across all samples, albeit to various abundances, confirming that most adults are colonized by *C. acnes*, *Corynebacteria* and *Staphylococci* [10, 17] (Supplementary Fig. S1A) (Supplementary Tables S1 and S2). In each volunteer, 2 to 9 species (median 4 species) belonging to 6 to 2 genera (median: 3 genera) accounted for the majority of the biomass (90% of sequenced 16S rRNA gene), as observed by Escapa and colleagues [37]. The remaining biomass was attributed to diverse species (18 to 51, median 24 species). Community structure analyses showed distinct compositions, and ten out of eleven microbiomes could be assigned to a community state type (CST) [9, 10] number according to the dominance of *Corynebacterium* spp. (CST5, *n* = 6), *S. epidermidis* (CST3, *n* = 2), *S. aureus* (CST1, *n* = 1), and *Cutibacterium* spp. (CST4, *n* = 1). One microbiome showed comparable abundances of *Corynebacterium* spp., *Cutibacterium* spp., and *Staphylococcus* spp., preventing a clear assignment to a CST (Fig. 1B) (Supplementary Fig. S1B, C) (Supplementary Tables S1 and S2). This analysis revealed a high abundance of *S. aureus* in communities B and C (27.1% and 75.8%, respectively), and a low abundance in A and D (0.2% and 1.6%, respectively).

Culturomics were used to isolate the widest possible diversity of species (identified by MALDI-MS) and clones (initially distinguished by colony morphology) for each volunteer (Supplementary methods). This led to recovery of 228 bacterial isolates (from 16 to 29 per volunteer). The genomes of all strains were sequenced and analyzed (Supplementary Table S3). After identification of clonal strains, the collection comprised 119 unique strains of 33 different species. The most frequently isolated species were *C. acnes*, *S. epidermidis*, and *C. avidum*. Comparison of species detection by 16S rRNA gene sequencing and culturomics confirmed that *C. granulosum*, *C. accolens* and strict anaerobes were less frequently recovered, aligning with previous culture-based studies [16, 38–40] (Supplementary Fig. S1A). Notably, *C. acnes* and *D. pigrum*, which are often considered difficult to culture [16], were consistently recovered in our conditions. Seven to 13 species were recovered per volunteer, including the major species of the volunteers’ microbiomes (Fig. 1C), supporting previous observations that dominant nasal species are culturable [37]. Only two abundant species could not be isolated: *Corynebacterium macginleyi* (abundant in Volunteer D) and strict anaerobes such as *Delftia acidovorans* or *Peptoniphilus* spp. (both abundant in Volunteers I and J).

The intraspecies phylogeny of all isolates was assessed using the DSMZ Type Strain Genome Server [41]. In general, isolates of the same species were of clonal origin within an individual microbiome, but clonally unrelated across different microbiomes (Supplementary Table S3). However, we found a few exceptions. For instance, the volunteers B, E, F, J, and K were simultaneously colonized by two clonally distinct strains of *S. epidermidis.* Additionally, clonally identical strains were detected across multiple volunteers: 10 individuals carried the same *C. tuberculostearicum/“kefirresidentii”* [42] strain, and four carried the same *C. acnes* strain. These strains may have been transferred between the volunteers as they shared the same work environment.

### Nasal communities show different interaction networks

**Fig. 2:**
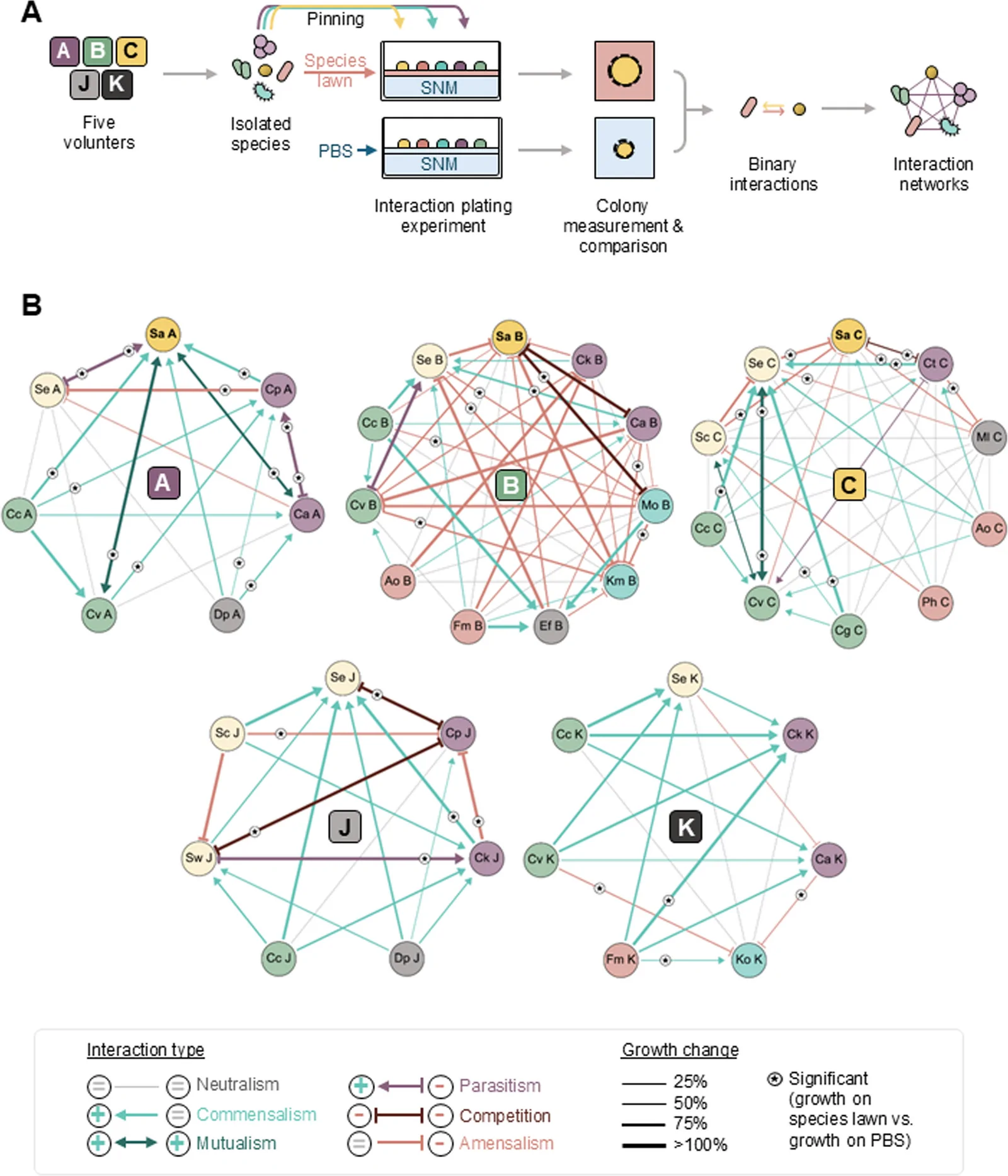
Binary interaction networks of the nasal communities **A, B, C, J, **and **K. A** Schematic methodology used to assess bacterial binary interactions within five communities. **B** For each community, interactions were determined for every pair of nasal strains on SNM agar and using colony area as a growth metric. Each strain pair was tested in both orientations (as background and as pinned species), and changes in colony area were used to define the type and strength of the interaction, as shown by the color, shape, and thickness of the edges. Interaction types were classified as follows: mutual (the two strains benefited from each other), commensal (one strain benefited while the other was unaffected), amensal (one inhibited, the other unaffected), competition (both inhibited), parasitic (one benefited at the expense of the other), and neutral (no detectable change). Missing edges indicate that the interaction could not be assessed due to limited detection of the pinned species. Each edge is based on three biological replicates. The interactions inducing a significant change in the colony area of at least one of the two involved strains are annotated with a star (*P*_adj_ < 0.05 two-way ANOVA with Dunnett’s correction). Nasal strain species are indicated in the nodes. Dark yellow: *Staphylococcus aureus* (Sa). Light yellow, other staphylococci: *Staphylococcus epidermidis* (Se), *Staphylococcus capitis* (Sc), *Staphylococcus warneri* (Sw). Green, *Cutibacteria*: *Cutibacterium acnes* (Cc), *Cutibacterium avidum* (Cv), *Cutibacterium granulosum* (Cg). Orange, anaerobic Gram-positives: *Anaerococcus octavius* (Ao), *Finegoldia magna* (Fm), *Peptoniphilus harei* (Ph). Verdigris, Gram-negatives: *Klebsiella michiganensis* (Km), *Klebsiella oxytoca* (Ko), *Moraxella osloensis* (Mo). Gray, other species: *Dolosigranulum pigru*m (Dp), *Enterococcus faecalis* (Ef), *Micrococcus luteus* (Ml). Purple, *Corynebacteria*: *Corynebacterium accolens* (Ca), *Corynebacterium “kefirresidentii”* (Ck), *Corynebacterium propinquum* (Cp), *Corynebacterium tuberculostearicum* (Ct). See also Supplementary Fig. S2 and S3. Primary colony measurements, growth fold-changes and statistical results are detailed in Supplementary Tables S10 and S11

We speculated that strains colonizing the same human nose might show particular reciprocal support or inhibition. To investigate this, we selected volunteers A, B, C, J, and K based on the composition of their nasal microbiota. The volunteers A, B and C were colonized by *S. aureus* to different degrees. Volunteer A exhibited low-level but consistent *S. aureus* colonization (as confirmed by a previous metagenomic study [43]), whereas volunteers B and C showed higher levels. In contrast, volunteers J and K showed no detectable *S. aureus* colonization. These five communities also varied in overall composition and were classified into four different CSTs, reflecting the diversity of nasal microbiota and degree of *S. aureus* carriage (Fig. 1B, C). First, we quantified the binary interactions between co-isolated strains from each of these communities. We used an agar-based assay described previously [44] on synthetic nasal medium (SNM), which reflects the key nutritional features of the nasal cavity [36]. Each strain was plated to form a lawn on SNM agar and incubated for 6 h to initiate metabolic activity and early growth, providing a physiologically active background. All other strains of the community were then pinned on top of the lawn, or on SNM agar without a lawn as a control. After incubation, the size of colonies formed by the pinned strains was measured and used to determine interactions (Fig. 2A) (Supplementary Fig. S2).

Using this approach, we tested a total of 452 binary interactions, of which 266 interactions could not be evaluated as the involved pinned strains failed to form detectable colonies on SNM agar or on the bacterial lawn. The remaining 186 interactions (from 28 to 55 per community) included both positive and negative interactions in every community, indicating that nasal strains from the same microbiota can substantially impact each other’s proliferation (Fig. 2B). The interaction network of each nasal community was found to be unique, and the proportion of positive and negative effects was variable across the different communities (Supplementary Fig. S3A). In our agar-based assay, interaction types were defined by changes in colony growth of the pinned species relative to monoculture: mutual (the two strains benefited), commensal (one strain benefited, the other unaffected), amensal (one inhibited, the other unaffected), competitive (both inhibited), parasitic (one benefited, one inhibited), and neutral (no change). The communities A, J, and K were characterized by a high proportion of commensal interactions (47.4%, 61.1%, and 64.7%, respectively), while amensalism was less frequent (10.5%, 16.7%, and 17.7%, respectively). As reported previously, we found that *Corynebacterium propinquum* inhibited *S. epidermidis* proliferation [45] in both communities A and J. In contrast, community B was dominated by amensal interactions (50%), while neutral interactions were most common in community C (36.8% of detected interactions) (Fig. 2B) (Supplementary Fig. S3A).

Nasal species are known to produce antimicrobial compounds [22, 24]. We therefore additionally assessed the presence of inhibition halos around pinned colonies as a complementary readout to determine whether diffusible antimicrobial activity could contribute to the interactions detected based on colony size. No inhibition halos were observed in our assay, providing no evidence for such inhibition mechanism under our experimental conditions.

### Interactions of *S. aureus* with nasal species are strain- and community-specific

The communities A, B, and C were *S. aureus*-positive based on 16S rRNA gene analysis and culturomics (Fig. 1B, C). The corresponding *S. aureus* strains were designated Sa A, Sa B, and Sa C, respectively. The only additional trait shared by communities A, B, and C was the presence of distinct strains of *S. epidermidis* and *C. avidum* (Supplementary Table S3). Interestingly, the interactions of *S. aureus* with other species differed substantially between the three communities. The proliferation of Sa A was enhanced by most members of the community A, including *S. epidermidis* and *C. avidum*. In contrast, we found Sa B and Sa C to be either inhibited or not affected by strains of community B and C, respectively (Fig. 2B) (Supplementary Fig. S3B and C). These findings indicate that even in this concise dataset, binary interactions with nasal species do not provide a consistent predictor of *S. aureus* relative abundance in the human nasal cavity.

**Fig. 3:**
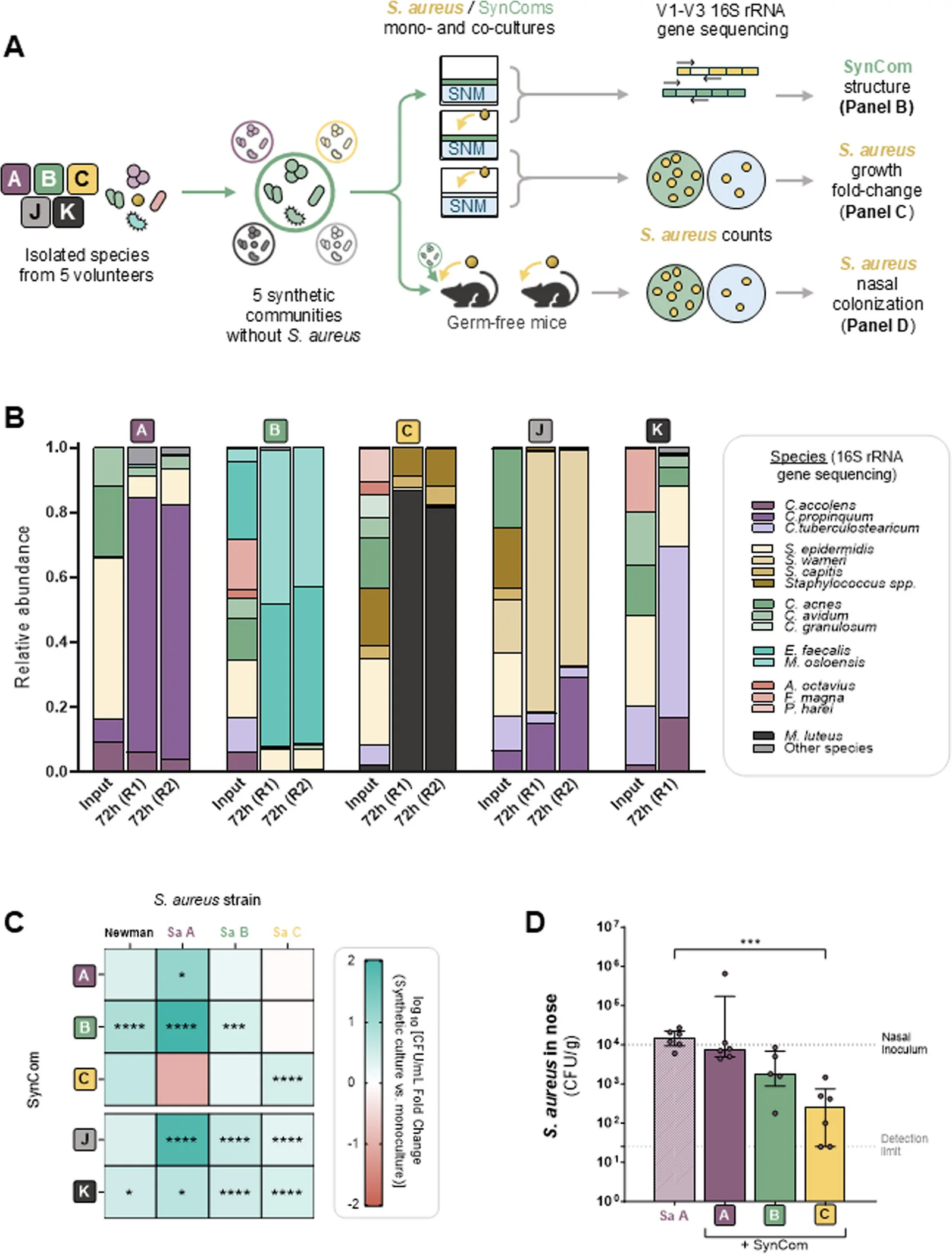
*S. aureus *and SynComs dynamics in mono- and cocultivation.** A** Schematic methodology used to study synthetic communities in vitro and in vivo. **B** Relative species abundance determined by 16S rRNA gene sequencing from frozen SynComs (Input) or SynComs cultivated 72 h on SNM agar in isolation for two biological replicates (R1, R2). Each color designates a species. Less abundant species were assigned to “Other species”. Complete data is detailed in Supplementary Table S4. See also Supplementary Fig. 4. **C** Heatmap depicting the growth fold-change induced by synthetic culture with five different SynComs (A, B, C, J, K) on SNM agar, for four *S. aureus* strains. Sa A, Sa B, and Sa C originated from volunteers A, B, and C, respectively. Positive fold-changes (verdigris) and negative fold-changes (red) indicate increases or decreases in *S. aureus* cell numbers in synthetic culture relative to the monoculture, respectively. Each fold-change is based on four biological replicates. Significant changes are marked with stars (*P < 0.05, **P < 0.01, ***P < 0.001, ****P < 0.0001, 2-way ANOVA). See also Supplementary Fig. S5. **D.** Barplots depicting the bacterial counts of *S. aureus* Sa A recovered from the noses of gnotobiotic mice, 7 days after nasal inoculation in isolation or in the presence of three different SynComs. Bars represent the median ± interquartile range from 5 to 6 animals per group. Dashed lines represent the nasal inoculum and the detection limit. Statistical significance is indicated by ***P_adj_ < 0.001 (Kruskal–Wallis with Dunn’s correction)

In complex communities, the outcome of pairwise relationships can be influenced by the presence of additional species, a phenomenon known as high-order interactions [34]. Therefore, we speculated that nasal communities may harbor a combination of traits that either promote or inhibit the proliferation of *S. aureus*. To assess this, we assembled five synthetic communities (SynComs) reflecting the species and strain composition of the microbiomes from volunteers carrying (A, B, and C) or not carrying *S. aureus* (J and K). SynComs were generated by mixing strains to equal optical densities, excluding the native *S. aureus* strain when present. We first evaluated the development of these SynComs on SNM agar, selected to mimic surface-attached growth in the nasal cavity (Fig. 3A). After 3 days of growth, community composition was analyzed using 16S rRNA gene amplicon sequencing. In the absence of *S. aureus*, each SynCom exhibited a distinct final structure, in general dominated by one or two species. For example, *C. propinquum*, *M. luteus and S. warneri* became dominant in SynCom A, C and J, respectively. This distribution differed from that observed in native communities, likely reflecting the simplified in vitro conditions of our SNM-based assay and the equal initial abundance of all community members at inoculation. However, most strains remained in detectable abundances, indicating that no major loss of community members occurred under these experimental conditions (Fig. 3B) (Supplementary Table S4). Addition of *S. aureus* (≈ 50% of the entire input) demonstrated that all tested *S. aureus* strains (Newman, Sa A, Sa B, and Sa C) were able to proliferate within all communities, but with strain- and community-dependent differences (Supplementary Fig. S4) (Supplementary Table S4). While general diversity of all communities was retained, final abundance of *S. aureus* strains differed markedly. In SynComs J and K, all *S. aureus* strains reached final abundances of 80% to 94% with only minor variations between *S. aureus* strains. In contrast, lower and more variable *S. aureus* abundances were observed in the other communities, varying from 75% (Sa C in SynCom B) to less than 10% (Sa A in SynCom C). Overall, SynCom C appeared the least permissive to *S. aureus* proliferation (47% mean *S. aureus* abundance), followed by SynCom B and SynCom A (54 and 64%, respectively). However, the permissiveness of these communities varied depending on the *S. aureus* strain, with the strongest variations observed for Sa A (Supplementary Fig. S4) (Supplementary Table S4).

Consistent with this, CFU determination of *S. aureus* in mono- and coculture showed SynComs J and K to generally enhance *S. aureus* final cell counts, suggesting that communities derived from *S. aureus*-negative volunteers do not inherently inhibit the pathogen’s proliferation under our experimental conditions (Fig. 3C) (Supplementary Fig. S5). In contrast, we found the reference strain Sa Newman to be mildly but significantly supported by SynCom B. The two other SynComs had a positive but non-significant impact on Sa Newman’s growth in synthetic culture. Sa B and Sa C showed slight growth benefits from the SynComs B and C, respectively, but were hardly affected by the other ones. Sa A showed the strongest responses, with its proliferation being increased in the presence of communities A and B, but limited by community C (Fig. 3C) (Supplementary Fig. S5).

To assess whether contact-dependent mechanisms or secreted products mediated the observed effects, we cultivated *S. aureus* Newman, Sa A, Sa B, and Sa C in 50% spent culture supernatants of the SynComs. Prior to filtration, each SynCom culture showed proliferation and contained final CFU counts between 2 x 10^7^ and 2 x 10^8^ (Supplementary Fig. S6A, B). The supernatant of the SynComs J and K significantly enhanced the growth of all *S. aureus* strains in a similar pattern to the one observed in the synthetic culture experiments on SNM agar (Supplementary Fig. S7A, B). Importantly, Sa A was also strongly supported by the supernatants of the SynComs A and B, whilst not growing in the spent medium of the SynCom C (Supplementary Fig. S7C). In contrast, Sa B and Sa C were not affected by the supernatant of the SynComs A, B, and C.

To evaluate whether these effects could be attributed to antimicrobial production, we analyzed the genomes of SynCom members using antiSMASH (Supplementary Table S5). A total of 183 biosynthetic gene clusters (BGCs) were identified across the SynComs (excluding *S. aureus*), ranging from 28 (SynCom A) to 45 (SynCom B). All communities included strains harboring BGCs allowing the production of compounds with demonstrated or potential activity against *S. aureus*, either through direct inhibition (e.g., gallidermin or ε-poly-L-lysine) [46, 47], or by facilitating competition for nutritional ions (e.g., staphyloferrin and other siderophores) [30] (Supplementary Table S5). However, the patterns of encoded BGCs did not correlate with observed supportive or inhibitory effects of these SynComs, suggesting that these compounds are unlikely to be the primary driver of community dynamics under these conditions. Altogether, our experiments suggest that diverse communities create an environment fostering or impairing *S. aureus* proliferation in a strain-specific manner. The effects appear to be dependent on nutrients consumed or produced by the communities rather than on secreted antimicrobial compounds.

To determine whether these effects are relevant in vivo, we developed a nasal colonization model using germ-free mice and tested the impact of SynComs A, B, and C on the proliferation of Sa A (Fig. 3C, D). In the absence of other nasal species, 1,5 x 10^4^ CFU/g of Sa A could be recovered from the noses of the mice after 7 days of colonization. Importantly, despite the presence of nasal strains that are expected to compete for space, adhesion sites and diverse complex nutrients, the nasal bacterial loads of Sa A were maintained in the presence of SynCom A and B but were reduced by two orders of magnitude in the presence of SynCom C (Fig. 3D). Taken together, these results show that the impact of nasal species and synthetic consortia on *S. aureus* is highly strain-specific, preventing the identification of a general pattern. Nasal communities can either support or inhibit *S. aureus* in a strain-dependent manner, thereby impacting colonization efficiency in context of communities. Sa A represented the most affected isolate in our collection.

### Communities affect the amino acid composition of the nasal environment differently

**Fig. 4:**
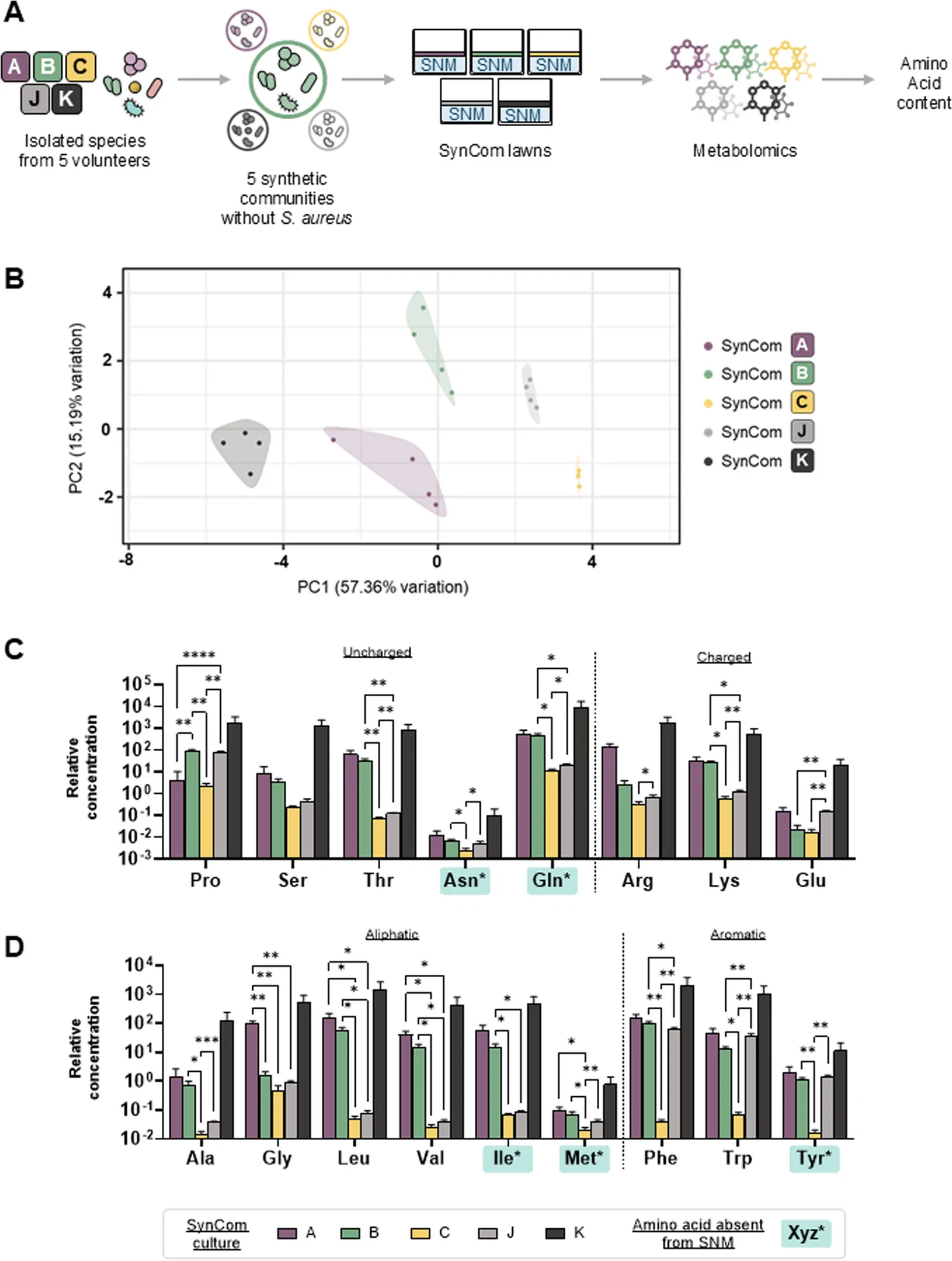
Amino acid concentrations detected in SynCom culture extracts.** A** Schematic methodology used to study the metabolome of synthetic communities*.* **B** Principal component analysis based on 17 amino acids detected in methanol extracts recovered from the five SynCom lawns after 3 days of cultivation on SNM agar. Each dot is based on the LC–MS data from one biological replicate (four per SynCom). Ellipses represent the 95% confidence interval of each group. Group differences and dispersion were assessed using PERMANOVA (*P* = 0.001, *R*^2^ = 0.8974), and PERMDISP (*P* = 0.005). **C, D** Bar plots showing the relative concentration of polar (**C**) and nonpolar (**D**) amino acids detected in methanol extracts recovered from the five SynCom lawns (A, B, C, J, K) after 3 days of cultivation on SNM agar. Cysteine and histidine were not assessed due to analytical limitations, and aspartic acid was not detected in the samples. Amino acids marked with a star and highlighted in light blue are not present in the original composition of SNM. Relative concentrations were calculated using a ^13^C-labeled internal standard, and further normalized to the particles extracted from the respective community, as measured by optical density of the methanol extract. Bars represent the mean relative concentration ± SD from four biological replicates, represented as log_10_-transformed values. Statistical significance is indicated by *P_adj_ < 0.05, **P_adj_ < 0.01, ***P_adj_ < 0.001, ****P_adj_ < 0.0001 (two-way ANOVA with Tukey’s correction). See Supplementary Table S6

Amino acids are essential for bacterial growth. Nasal secretions are devoid of asparagine, aspartate, glutamine, isoleucine, methionine, and tyrosine, and these are, therefore, not present in SNM [36]. We speculated that changes in amino acid availability might explain the effects of nasal communities on *S. aureus*. To investigate this, we compared the amino acid profiles of methanol extracts recovered from SynComs lawns after 3 days of growth on SNM agar (Fig. 4A) (Supplementary Table S6). Principal component analyses showed each community to have a distinct impact on amino acid availability (Fig. 4B). Five of the six amino acids originally absent from SNM were detected in all SynCom cultures, suggesting that nasal communities have the potential to release (actively or passively) these molecules and therefore enhance the nutrient diversity in the medium (Fig. 4C, D). SynCom K induced the highest amino acid concentrations, whereas SynCom C and J showed generally lower levels (Fig. 4C, D). Additionally, SynCom C was characterized by reduced concentrations of the aromatic amino acids tyrosine, tryptophan, and phenylalanine compared to all other communities (Fig. 4D). It remains unclear whether the different amino acid levels in SynCom extracts were caused by active release from living cells by metabolic overflow or represent different levels of cell lyses. However, all communities exhibited either substantial growth (B, C, J) or stable CFU counts over 3 days (A, K) (Supplementary Fig. S6C), a pattern that does not align with amino acid profiles. This suggests that lysis of the inoculum is not solely responsible for the observed release of amino acids. Altogether, our findings demonstrate that the growth of nasal microbial communities alters the nutritional composition of SNM and suggest that SynCom C may impose a particularly strong nutritional stress on *S. aureus*. Whether these nutritional differences contribute to the reduced growth of Sa A observed in SynCom C both in vitro and in vivo remains to be determined.

### Tyrosine auxotrophy impacts *S. aureus* A proliferation in the context of community products

To determine whether amino acid profiles shaped by the communities affect *S. aureus* proliferation, we explored the relevance of amino acid availability and its effects on diverse *S. aureus* strains. Only Sa A showed a growth defect in SNM but proliferated in rich medium (Supplementary Fig. S8). Supplementing SNM with the six lacking amino acids in a mixture or individually showed tyrosine to be necessary and sufficient to restore the growth of Sa A in a dose-dependent manner (Supplementary Fig. S9). This suggested Sa A to be auxotrophic for tyrosine.

**Fig. 5:**
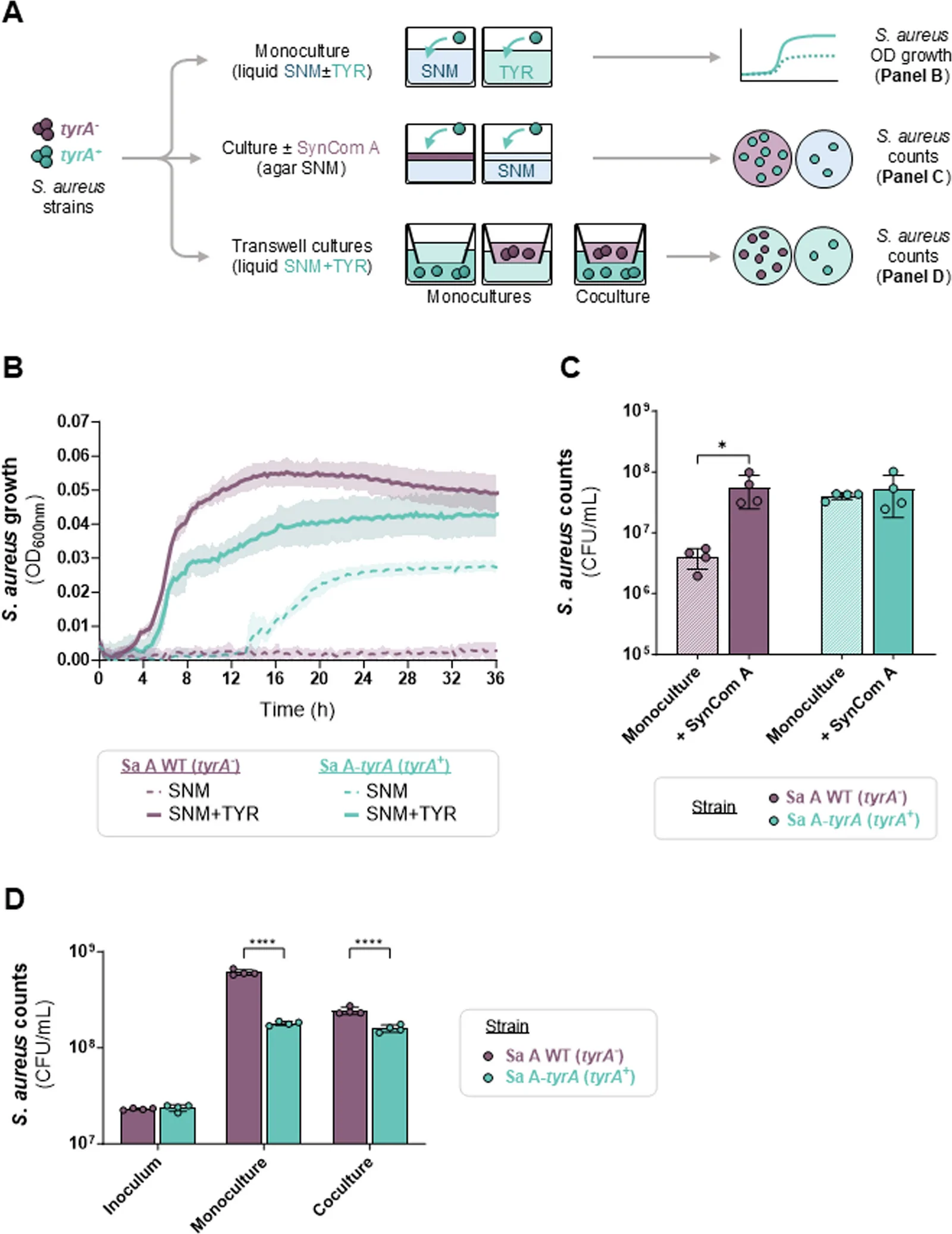
Effect of *tyrA* impairment on the proliferation of *S. aureus* Sa A in SNM. For all panels: the nasal *S. aureus* strain with an impaired *tyrA* gene (Sa A WT) is referred as *tyrA*^−^ and shown in purple, while the strain with the restored *tyrA* gene (Sa A-*tyrA*) is named *tyrA*^+^ and shown in verdigris. Lines or bars represent mean ± SD of four biological replicates. Statistical significance is indicated by *P_adj_ < 0.05, ****P_adj_ < 0.0001 (two-way ANOVA with Šídák’s correction). **A** Schematic methodology used to study the behavior of *S. aureus tyrA*^−^ and *tyrA*^+^ strains. **B** OD_600nm_ growth kinetics in SNM and SNM + 100 µM tyrosine (TYR). See also Supplementary Fig. S11A, C and E. **C** Barplots showing the bacterial counts of *S. aureus* cultivated on SNM agar in isolation or in the presence of the SynCom A over 3 days. See also Supplementary Fig. S11B, D. **D** Barplots showing the bacterial counts of *S. aureus tyrA*^+^ and *tyrA*^−^ in the inoculum, cultivated in SNM + 100 µM tyrosine either in isolation or cocultured with each other over 24 h using the transwell system. See also Supplementary Fig. S12

Whole-genome sequencing of Sa A revealed a frameshift mutation (T insertion at position 817/1093) in *tyrA*. This gene encodes a chorismate mutase/prephenate dehydrogenase, an enzyme shown to be essential for tyrosine biosynthesis in *Bacillus*, *E. coli*, and other species [48, 49] (Supplementary Fig. S10). Genomic reversion of the frameshift mutation in Sa A enabled growth in SNM (Fig. 5A, B). The growth-enhancing effect of the SynCom A on Sa A was abrogated when the frameshift in *tyrA* was removed, indicating that this mutation caused dependency of the strain on tyrosine released (actively or passively) by the community (Fig. 5C). Introduction of Δ*tyrA* mutations in *S. aureus* Newman and USA300 JE2 induced tyrosine auxotrophy and community-dependency, as observed for Sa A. Phenotypes of Δ*tyrA* mutants were rescued by plasmidic complementation (Supplementary Fig. S11). Altogether, these findings show *tyrA* deficiency to cause tyrosine auxotrophy in Sa A*,* making its growth dependent on tyrosine availability and enhanced by the presence of SynCom A under the nutritionally poor conditions of SNM.

### Tyrosine auxotrophic strains exhibit a fitness advantage in the presence of tyrosine

Reduction of metabolic labor offers auxotrophs a fitness advantage when they acquire the missing amino acid from an external source [50, 51]. To assess this in *S. aureus*, we conducted mono- and cocultures of Sa A WT strain (*tyrA*^−^) and the *tyrA-*restored mutant (*tyrA*^+^) in SNM + tyrosine using a transwell system that allows nutrient diffusion between separately growing strains (Fig. 5A, D). In the presence of tyrosine, Sa A WT reached higher cell densities than the *tyrA*-restored mutant, both in monoculture and when strains competed for the shared nutrients (Fig. 5D) (Supplementary Fig. S12A). Similar effects were observed in the Sa Newman background during coculture, and in the Sa JE2 background in monoculture (Supplementary Fig. S12), suggesting that tyrosine auxotrophy provides a general selective advantage when the amino acid is supplied by external sources.

### Nasal* S. aureus* strains exhibit various amino acid requirements

**Fig. 6:**
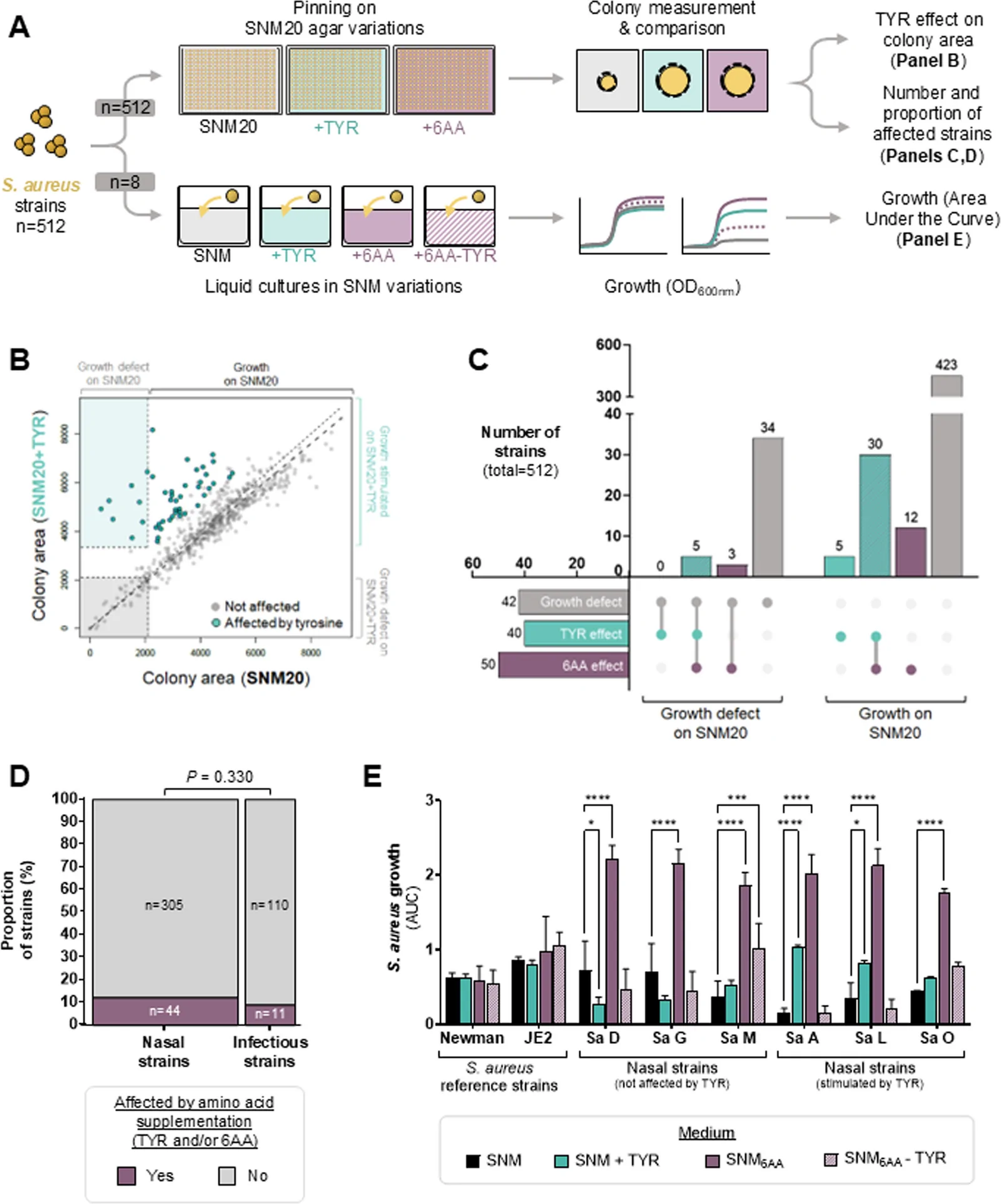
Phenotypic tyrosine dependency of *S. aureus* isolates*. ***A** Schematic methodology used to identify phenotypic auxotrophies in *S. aureus* strains. See also Supplementary Fig. S13A–C. **B** Dotplot depicting the effect of tyrosine (TYR, 100 µM) supplementation on the growth on SNM20 of a collection of *S. aureus* strains (*n* = 580 depicted colonies; *n* = 525 unique isolates). Colored zones denote thresholds that categorize isolates with growth defects on SNM20 that can either be restored (light blue zone) or not (grey zone) by tyrosine supplementation. The diagonal dashed lines represent the linear regression corresponding to a ratio of one (thin line) or the actual linear regression (thick line), showing that most *S. aureus* were not affected by tyrosine supplementation. Strains showing a significant growth improvement in the presence of tyrosine are highlighted in verdigris (*P*_adj_ < 0.05, t-test with Bonferroni correction). Each dot represents the colony area from three biological replicates. **C** Upset diagram showing responses to amino acid supplementation across 512 *S. aureus* strains*.* Strains from human origin and unique clonality were selected from the screening shown in Panel A. Horizontal bars represent the number of strains exhibiting a growth defect on SNM20 agar, or a growth stimulation by supplementation with either the six amino acid mix (6AA, 100 µM each) or tyrosine (TYR, 100 µM) alone. Vertical bars indicate the number of strains showing each growth phenotype, sorted by their basal ability to form colonies on SNM20 without amino acids. **D** Mosaic plot showing the proportion of *S. aureus* human strains not affected or being stimulated by amino acid supplementation (tyrosine and/or the six amino acid mix), according to their isolation site. Nasal strains were obtained from the nose of healthy volunteers, whereas infectious strains were derived from non-nasal human sources and associated with a reported case of *S. aureus* infection. Strains with undefined isolation site or no growth under any of the tested conditions were excluded from the analysis. The area of each tile is proportional to the number of strains with the corresponding phenotype, as indicated by the numbers within tiles. Statistical significance was assessed using Fisher’s exact test. **E** Bar plots showing the proliferation of 8 *S. aureus* strains in SNM or in SNM supplemented with tyrosine (TYR), with a mixture of the six amino acids missing from SNM (6AA), or with the same mixture from which tyrosine was removed (6AA-TYR). 100 micromolar of each amino acid was used. Each bar displays the mean AUC + SD from three biological replicates of growth kinetics. Statistical significance is indicated by *P_adj_ < 0.05, ***P_adj_ < 0.001, ****P_adj_ < 0.0001 (two-way ANOVA with Dunnett’s correction)

It remained unclear whether tyrosine auxotrophy and the corresponding dependence on certain producer communities represent an isolated observation, or if it reflects a more widespread phenomenon, impacting *S. aureus* proliferation in the context of nasal communities. We examined the frequency of frameshift mutations in the *tyrA* gene across 9340 *S.* *aureus* genomes and found the gene to be potentially disrupted in ≈1% of cases (n = 98 genomes). However, phenotypic auxotrophy seemed more informative because the altered primary structure of TyrA, along with mutations in other genes of the pathway or in its regulatory network, might also cause auxotrophy. We gathered a biobank of 512 *S. aureus* human strains and assessed their growth on standard SNM and SNM supplemented with tyrosine (Fig. 6A) (Supplementary Fig. S13A–C). Forty-two strains (8.2%) failed to proliferate on SNM. Supplementation of the medium with tyrosine rescued the growth of five of them, and three additional strains formed colonies only in the presence of the six amino acids missing from SNM (Fig. 6B, C). However, among strains that were able to grow on plain SNM, we found 47 (9.2% of all strains) whose proliferation was significantly enhanced in the presence of tyrosine or the six amino acid mixture (Fig. 6B, C) (Supplementary Fig. S13D, E). In total, 55 of the 512 tested *S. aureus* strains (10.7%) were stimulated by amino acid supplementation, with tyrosine alone promoting growth in 40 strains (7.8% of all strains, 8.2% of nasal strains), indicating strain-specific nutritional requirements. When focusing on strains with well-defined isolation sites, we found similar ratios of strains showing amino acid dependency among nasal strains and those from infective foci (Fig. 6D). Together, these findings suggest that *S. aureus* strains show varying degrees of amino acid dependency, including complete auxotrophy, which may influence their integration into certain microbial communities during colonization.

**Fig. 7:**
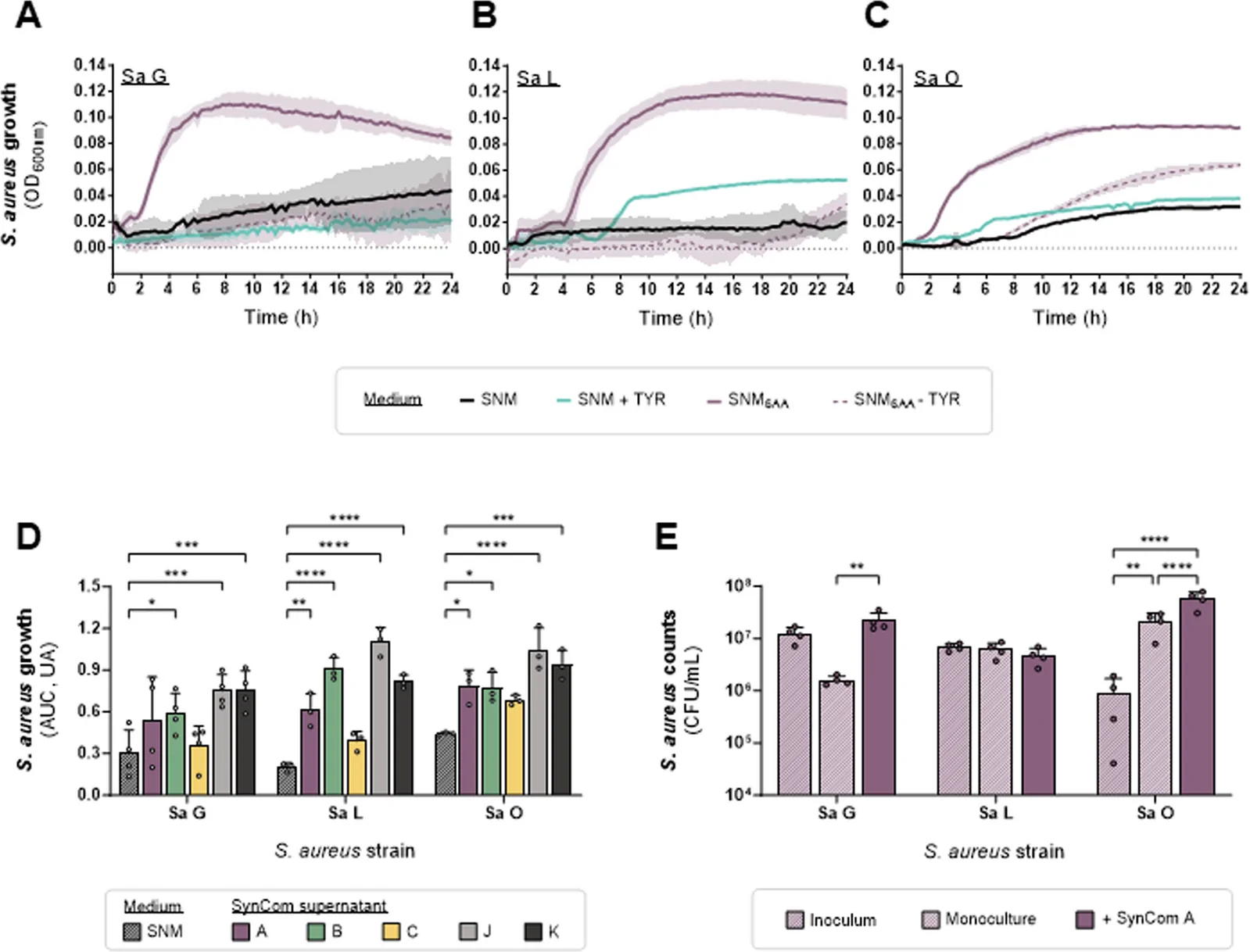
Growth behavior of nasal *S. aureus *strains with amino acid dependencies.** A**, **B**, **C** Proliferation of Sa G (**A**), Sa L (**B**) or Sa O (**C**) in SNM or in SNM supplemented with tyrosine (TYR), with a mixture of the six amino acids missing from SNM (6AA), or with the same mixture from which tyrosine was removed (6AA-TYR). 100 micromolar of each amino acid was used. Lines represent the mean ± SD of three biological replicates of OD_600nm_ measurements, taken every 15 min over 24 h. **D** Proliferation of Sa G, Sa L, or Sa O in SNM or in the presence of 50% concentrated spent culture supernatant of the SynComs A, B, C, J and K. Each bar displays the mean AUC + SD from three to four biological replicates of growth kinetics. Statistical significance is indicated by *P_adj_ < 0.05, **P_adj_ < 0.01, ***P_adj_ < 0.001, ****P_adj_ < 0.0001 (two-way ANOVA with Dunnett’s correction). **E** Barplots depicting the bacterial counts of Sa G, Sa L or Sa O in the inoculum, and after incubation for 3 days in monoculture and in presence of the SynCom A on SNM agar. Bars represent the mean + SD of four biological replicates. Statistical significance is indicated by **P_adj_ < 0.01, ****P_adj_ < 0.0001 (two-way ANOVA with Šídák’s correction)

To further validate this, we selected five nasal strains that responded differently to tyrosine supplementation in our initial screen and cultured them in liquid SNM with various amino acid combinations (Fig. 6E). Tyrosine supplementation enhanced the proliferation of Sa O and Sa L, but not that of Sa D, Sa G, and Sa M. The growth of these strains was rescued in the presence of the six amino mix, but only when tyrosine was included (Figs. 6E and 7A–C). This suggests that tyrosine is necessary but not sufficient for these strains to grow in SNM, potentially due to multiple amino acid auxotrophies. Proliferation of Sa G, Sa L, and Sa O in SNM was enhanced by SynCom supernatants or living cells, largely reflecting the behavior of Sa A (Fig. 7D, E). Genome analysis showed the *tyrA* gene of Sa G to be disrupted by a frameshift mutation (T deletion in position 325/1091) (Supplementary Fig. S10). In contrast, no evident detrimental *tyrA* mutations were identified in the remaining strains, suggesting that other mutations or regulatory mechanisms may underlie their dependency on amino acid supplementation. Collectively, these results show that amino acid requirements are strain-specific among nasal *S. aureus* isolates, with phenotypic tyrosine auxotrophy being regularly identified among human isolates.

### Tyrosine auxotrophy impacts *S. aureus* proliferation in the context of nasal species in vivo

**Fig. 8:**
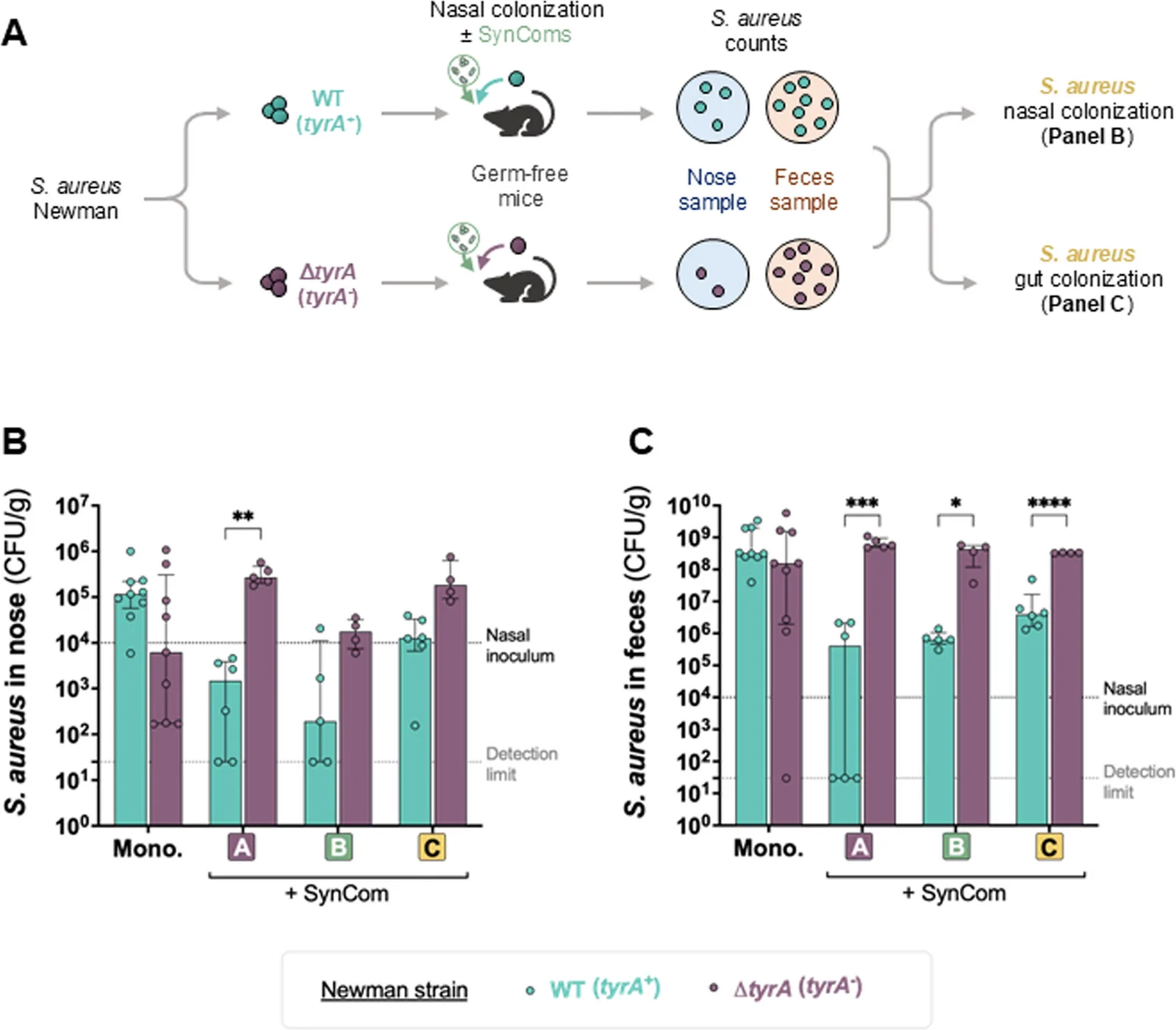
Effect of *tyrA* deficiency on the ability of *S. aureus *to colonize the nasal cavities and the intestine of gnotobiotic mice.** A** Schematic methodology used to study the effect of tyrosine auxotrophy in vivo. **B**, **C** Barplots depicting the bacterial counts of *S. aureus* Newman WT (verdigris) and ∆*tyrA* (purple) recovered from the noses (**B**) or feces (**C**) of gnotobiotic mice 7 days after nasal inoculation in solitude (Mono.) or in the presence of SynCom A, B, or C. Bars represent the median CFU per gram of organ ± interquartile range from 4 to 9 animals per group. Dashed lines represent the nasal inoculum and the detection limit. *P_adj_ < 0.01, **P_adj_ < 0.05, ***P_adj_ < 0.001, ****P_adj_ < 0.0001 Welch’s* t*-tests (WT vs. mutant) with Holm–Šidák correction

We used the *S. aureus* strain Newman along with its isogenic Δ*tyrA* mutant to assess the relevance of tyrosine biosynthesis in context of nasal communities in vivo (Fig. 8A). When colonizing the animals in solitude, the WT strain (*tyrA*^+^) reached a median colonization density of 10^5^ CFU/g of nose, exceeding that of Sa A by one order of magnitude (Figs. 3D and 8B). This is in line with the general recognition of Newman as good nasal colonizer of murine models [30, 36]. Introduction of the Δ*tyrA* mutation resulted in a strongly increased variability, with only some mice showing strongly reduced CFU counts compared to the WT strain (Fig. 8B). This effect was mitigated when *S. aureus* was enumerated in murine feces, possibly reflecting higher nutrient availability within the gut (Fig. 8C). Interestingly, during co-colonization with synthetic communities, the *tyrA*-deficient strain reached significantly higher cell counts than the WT strain. The effect was apparent for the three communities but was strongest for SynCom A (~ 180-fold increase) and less pronounced for SynCom C (~ 15-fold increase). Similar dynamics were observed in the mice feces (Fig. 8C).

Together, these data indicate that presence of nasal species infers a competitive pressure on *S. aureus*, and that the inactivation of *tyrA* improves the bacterium’s fitness in this context. One possible explanation is that the presence of nasal species alters tyrosine availability within both the nasal and intestinal cavities. While this effect may result from tyrosine release from community members, this is most likely not the only explanation, as SynCom C was shown to have an inhibitory potential and to produce only low levels of tyrosine in vitro (Fig. 4D). It seems possible that the community also impacts tyrosine availability indirectly, for example by degrading mucins, damaging host epithelial cells or by interfering with *S. aureus* production of cell-targeting toxins. Further experiments are needed to test these hypotheses.

Nonetheless, our data strongly suggest that amino acid availability in the nasal cavity is impacted by nasal communities in vivo and can shape *S. aureus* colonization in a strain and community-dependent fashion.

## Discussion

Approximately one-third of the human population is persistently colonized by *S. aureus,* while the remainder carry the pathogen only transiently [8]. Host genetics appears to play only a minor role in this process [9], and increasing evidence suggests that the nasal microbiome significantly impacts *S. aureus* colonization. In particular, direct interference by nasal species producing antibacterial molecules to kill *S. aureus* has been described [21, 22, 24]. However, while such antimicrobial molecules can potently impact microbial community composition, they alone are insufficient to explain the complex and variable dynamics of *S. aureus* colonization between and within individuals. Besides direct interference, competitive or collaborative consumption of available nutrients is known to govern structures of both environmental and host-associated bacterial microbiota [52]. Within communities, competitive or collaborative interactions are largely dependent on accessible nutrients and the metabolic needs of the associated strains. Accordingly, nutritional interactions within human microbiomes are expected to differ between different body sites as nutritional conditions are highly variable. The interactions occurring between nasal species and *S. aureus* within the nutritionally limited environment of the human nose remain poorly understood.

In our experiments, we isolated nasal bacterial communities from eleven human volunteers. While this cohort is small, the individuals were chosen to represent different community state types [9, 10] and *S. aureus* carriage levels. The resulting strain collection includes all dominant taxa present in the microbiomes of healthy adults and differs from those previously established, which often included individuals from clinical settings [16] or were biased towards the isolation of aerobic [18, 39, 53, 54] or clinically relevant taxa [20–23, 38, 40, 55]. Additionally, it captures the intraspecies diversity, which can now be further explored using the LaCa collection. Only some strict anaerobes and a few species that were extremely low in abundance according to 16S rRNA analysis could not be isolated and cultured. We cannot exclude that these species, along with other isolates showing a growth defect in SNM, might engage in direct or higher-order interactions influencing *S. aureus* proliferation. Future work should therefore focus on improving cultivation methods for fastidious nasal species, including optimized media or more complex culture systems such as host-associated models (e.g., air–liquid interface, organoids or nose-on-chips) [56–58], to better recapitulate the nasal environment. In this context, refining the composition of SNM could be valuable, as its original formulation was established in 2014 using metabolomic approaches that have since evolved considerably and was based on a limited number of individuals.

Our generated interaction networks clearly demonstrate diverse and strain-specific interactions between *S. aureus* and nasal species, suggesting that *S. aureus* strains may be adapted to distinct niches within specific communities. However, the contribution of individual strains and the factors they may secrete remain to be clarified in our conditions. For instance, *M. luteus* has been reported to inhibit *S. aureus* virulence and growth through the secretion of bioactive compounds [59, 60], a mechanism that may contribute to the inhibitory potential of the SynCom C where it dominated in our experiments. However, when binary interactions were assessed, *S. aureus* inhibited *M. luteus* and not vice versa. In contrast, the supportive effect of SynCom B on *S. aureus* may be related to its high abundance of *E. faecalis*, which has been shown to engage in cooperative interactions with *S. aureus *[61, 62]. Dissecting the role of individual species through bottom-up or top-down approaches will be essential to better understand these community dynamics, an area of growing interest in nasal microbiome research [27, 63].

While we currently lack a precise understanding of the relevant metabolites underlying many of these interactions, our analysis shows that collaborative usage of limited resources may represent an important trait within the nasal ecosystem. We identify commensalism (positive effects of one species towards another) as the most common type of interaction among nasal bacteria, dominating four out of five interaction networks in our study. In contrast, competition is described as the dominant type of interaction in gut and soil microbiota, where it is thought to play a greater role in stabilizing community structures than positive interactions [35, 64]. This difference may stem from the distinct nutritional conditions of the nasal cavities and suggests that bacterial interactions—and, by consequence, colonization resistance—are driven by different processes. In the nutrient-rich environment of the human intestinal tract, complete consumption of carbohydrates by resident bacteria has been shown to prevent the establishment of invading pathogens [1, 65, 66]. In contrast to the intestine, the nasal environment lacks complex carbohydrates but instead contains micromolar concentrations of carbon and energy sources such as glucose, succinate and pyruvate, along with a limited selection of proteinogenic amino acids [36]. Under these conditions, we demonstrated that nasal communities vary in their amino acid profiles and therefore in their ability to release or consume amino acids, which may contribute to their differential effects on *S. aureus* proliferation both in vitro and in vivo. Strikingly, we found that the interactions between *S. aureus* and nasal species were highly strain-specific with only few conserved patterns at the species level. This suggests that reciprocal adaptation between strains coexisting within a single individual may play a more critical role than broader species-specific traits. It is well known that targeted exchange of central metabolites between microbial strains and species frequently occurs, since “division of labor” provides fitness benefits for the participants and contributes to community diversity and long-term stability [67–71]. However, the precise mechanism underlying the excretion of central metabolites has not been thoroughly investigated in our assays. The prevailing hypothesis is that metabolites are released as consequence of metabolic overflow [72, 73]. This phenomenon has primarily been described for major fermentation products (e.g., lactate, acetate, and ethanol), but it also encompasses the secretion of primary metabolites such as amino acids and nucleosides [73–75]. Strain-specific secretion of isoleucine and other organic acids attributed to overflow metabolism has been reported for *S. aureus *[76]. Intracellular biotin is released not only by *S. aureus* but also by nasal strains and amounts are strain and species-dependent [77], supporting the notion that multiple intracellular metabolites can be externalized. However, metabolomic analyses suggest that the presence of all secreted intracellular metabolites cannot be fully explained by metabolic overflow alone [78]. Alternative molecular mechanisms contributing to externalization may involve transport via facilitated diffusion through non-specific mechanosensitive channels [79], as well as compromised membrane integrity and cell lysis [80]. It appears most plausible that extracellular amino acid levels in our experiments result from a combination of these mechanisms, which are challenging to disentangle. Accordingly, our observation that nasal communities have discrete enriching or depleting effects on nutrient availability is in line with observations of others and ecological concepts.

In our screen, the strain Sa A was isolated from a community providing high amounts of tyrosine. This strain exhibited tyrosine auxotrophy and depended on communities producing this amino acid to thrive in vitro. We found tyrosine auxotrophy to provide a fitness benefit to Sa A over its prototrophic counterpart in the presence of exogenous tyrosine. Auxotrophs are common in bacterial communities and impact their diversity and stability, often through active cross-feeding between individuals with different metabolic requirements [67, 70]. Although our experiments do not allow to determine whether tyrosine release by the SynComs is an active process, it is tempting to speculate that it may be. Similarly, *S. aureus* Sa A might release certain metabolites, as it enhanced growth of the nasal species *C. avidum* and *C. accolens* in our binary interaction screen. Nevertheless, additional experiments are required to confirm the presence of active cross-feeding between *S. aureus* and nasal species. In any case, the growth of SynComs clearly alters nutrient availability in specific ways. In this context, the emergence of auxotrophic mutants aligns with the ecological “Black Queen” hypothesis, which describes adaptive gene loss, associated “cheater” phenotypes and resulting metabolic dependencies between strains in microbial communities sharing public goods [81, 82]. Altogether, these observations suggest that *S. aureus* Sa A may have developed as an adaptation towards an environment that provided sufficient amounts of tyrosine, most likely shaped by the specific composition of community A.

This phenomenon is likely relevant in vivo*,* as our gnotobiotic animal experiments showed that a *tyrA*-deficient strain of *S. aureus* Newman reached higher cell densities in the presence of diverse nasal communities compared to its prototrophic counterpart. However, it should be noted that this is the first description of a gnotobiotic nasal colonization model, which therefore has several limitations and requires careful interpretation. The current setup allows for *S. aureus* enumeration but does not accurately capture the community composition within the nares of animals. As a result, the community stability over time remains unclear, and it is not known if the metabolic fingerprints identified in vitro are replicated in vivo. Moreover, the overall variability of colonization, the characteristic behaviors of the bacterial and mouse strains, and the reproducibility of results across different facilities and researchers has yet to be determined. We assume that nutrient availability, colonization densities and community composition within the nasal cavity may be influenced by diverse factors, such as food and water sources, number of animals per cage and their social interactions. Our experimental groups are currently too small to reliably answer these questions, and additional datasets generated in diverse facilities worldwide are needed to identify and address the limitations of this model. Another limitation is the usage of *S. aureus* Newman as a model strain due to its known efficacy for colonization experiments. This strain is hypervirulent due to constitutive activation of the SaeRS-signaling regulon, resulting in increased production of adhesins and toxins that induce host cell damage [83–86]. It remains uncertain whether such phenotypes can mask community-mediated effects of nutrient competition to a certain extent in vivo*,* as they might increase nutrient release from damaged host cells. Finally, *S. aureus *in vivo colonization and relative abundance are certainly multifactorial and not solely driven by amino acid availability, as detailed below.

First, nutritional interactions in vivo might stretch beyond those tested in our in vitro conditions [87]. We have demonstrated before that competition for iron binding siderophores shapes interactions between *S. aureus* and nasal species [23, 30], a factor that was not investigated in the in vitro experimental setup of this study but might contribute in vivo. Additionally, host-derived complex nutrients must be considered. Mucins, for example, are abundant in the nasal cavity and provide a rich source of peptides and carbohydrates, but are not constituents of SNM. Mucin degradation by microbial communities is well-documented within the gut and the oral cavity, and has also been suggested to foster interactions between *Neisseria meningitidis* and *Streptococcus mitis* within the nasopharynx [88, 89]. The relevance of mucin degradation for interactions between nasal species and *S. aureus* remains to be investigated. Second, colonization efficiency will also depend on the ability of bacteria to adhere to the host epithelia. While the molecular mechanisms of *S. aureus* adherence to the nasal epithelium have been studied extensively [90], the adhesion mechanisms of other nasal species are largely unclear. It is therefore unknown if competition for adhesion sites influences *S. aureus* colonization dynamics in the nasal cavity. Third, the host immune system is known to impact *S. aureus* colonization. For example, immune stimulation by *S. epidermidis* has been suggested to reduce *S. aureus* colonization [17]. However, the extent of immune stimulation by diverse nasal communities remains unclear and was not assessed in our current experimental setup. Finally, direct interference by nasal species through the production of bacteriocins, quorum-quenching molecules, reactive oxygen species or antimicrobial lipids, may also shape *S. aureus* abundance but were not evaluated in this study. Accordingly, many factors will shape the proliferation of *S. aureus* within the nasal cavity. While we are only starting to understand the molecular mechanisms of interactions, their relative contributions to colonization density remain difficult to quantify.

We found that 8.2% of nasal *S. aureus* strains were stimulated by tyrosine supplementation, suggesting that the selection of decreased endogenous biosynthesis may occur repeatedly across diverse lineages. This observation raises the possibility that *S. aureus* strains in different volunteers adapt to the specific characteristics of their surrounding community, potentially improving long-term colonization and community stability. However, longitudinal sampling of *S. aureus* carriers, combined with repeated assessment of bacterial interactions and quantification of tyrosine biosynthesis by both *S. aureus* and communities, would be required to test this hypothesis.

Finally, it is important to assess if selection for auxotrophies within certain nasal communities might alter the ability of *S. aureus* strains to colonize different habitats or affect their infectivity. Despite possessing the biosynthesis genes, most *S. aureus* strains are phenotypically auxotrophic for proline, arginine and branched-chained amino acids. Acquisition of these amino acids from external sources influences *S. aureus* virulence and confers a competitive advantage during acute and chronic infections [91, 92]. Interestingly, arginine auxotrophy is thought to be selected in human colonizing strains, notably due to its abundance in nasal secretions [91]. Other strain-specific auxotrophies have also been reported for amino acids absent from the nasal environment, such as methionine, asparagine, and tyrosine [93], and several amino acid metabolic pathways were shown to be frequently affected by non-synonymous mutations in colonizing isolates [94]. In other ecosystems, and particularly in the intestinal niche, amino acid auxotrophs often emerge and foster cooperative interactions such as cross-feeding, which supports microbial diversity and community stability [67–69]. Nevertheless, the potential role of amino acid metabolism in *S. aureus* adaptation and interactions in the nasal niche is unclear. Our in vivo experiments suggested that tyrosine auxotrophy has a stronger impact on nasal than on intestinal colonization in the absence of other nasal species. A likely explanation is that the gut provides greater nutrient availability [95], which can compensate for the auxotrophy. However, in the presence of nasal communities, colonization dynamics in the nasal and intestinal niches became more similar, supporting the idea that some nasal species could establish and impose selective pressure on *S. aureus* in both environments. Investigating community dynamics across nasal and intestinal niches could provide valuable insight into their interconnection, a topic of growing interest in studies of *S. aureus* colonization [96, 97]. Direct comparisons of prototrophic and auxotrophic strains regarding infectivity fall beyond the scope of this manuscript. However, we found similar frequencies of tyrosine dependency in nasal isolates and those derived from infection sites. Additionally, mutants deficient in tyrosine biosynthesis were not identified as attenuated in transposon-sequencing screens in models of osteomyelitis [98], skin and soft tissue infections [99, 100], bloodstream and kidney infections [101], or growth in sputum of cystic fibrosis patients [102]. This suggests that tyrosine biosynthesis plays a limited role during invasive infection. A recent genomic study found the metabolic pathways of tryptophan, phenylalanine, and tyrosine to be more frequently affected by non-synonymous mutations in *S. aureus* isolates from colonization compared to those from infections [94]. If these mutations have functional consequences remains elusive. Dissecting the tyrosine biosynthetic pathway and its regulation in *S. aureus* would provide deeper insights into its role in shaping the bacterium’s physiology and microbial interactions during both colonization and infection.

In summary, our study shows that the ability of *S. aureus* to colonize the nasal ecosystem and to interact with its bacterial members is highly dependent on community- and strain-specific features. By combining community-based interaction assays with metabolic profiling, we provide new insights into nutritional interactions influencing bacterial dynamics within nasal communities. Among these, we identified provision of amino acids as one factor stimulating growth of certain *S. aureus* strains in the nasal microbial environment. However, additional and larger cohort studies are needed to further understand and control *S. aureus* persistence in the human nose.

## Methods

### Bacterial strains

The bacterial strains used in this study are listed in Supplementary Table S3 and Table S7. The nasal strains from the LaCa collection were isolated from the nose of eleven healthy adults. The volunteers A, B, D, E, F, G, H, I, and J had previously participated in a metagenomic study conducted in our laboratory [43], while the volunteers C and K were newly recruited. In all cases, participants were selected for the current study based on the following criteria: (1) a healthy status for at least 2 weeks prior to sampling, with no known illness or antibiotic treatment that could have influenced the nasal microbiota or immune status; (2) availability for daily sampling over three consecutive days; and (3) inclusion of both male and female participants, although full gender parity could not be achieved. The sample collection procedures were approved by the clinical ethics committee of the University of Tübingen (No. 109/2009 BO2) and informed written consent was obtained from the volunteers. Nasal samples were collected using sterile flocked swabs (eSwab, Copan) from the anterior nares of each volunteer. Briefly, one nasal swab per volunteer was serially diluted and plated on multiple rich agar media under aerobic, CO_2_-enriched (21% O_2_, 5% CO_2_), and anaerobic (Anaerocult® A, Merck) conditions at 37 °C for 3 to 10 days, following an isolation protocol of Kaspar et al. [16] and detailed in the Supplementary methods. Species were first identified from isolated colonies using matrix-assisted laser desorption/ionization–time-of-flight mass spectrometry [103], using Bruker Daltonik Biotyper (composite database; score cutoff ≥ 2.0) (Supplementary methods). To maximize clonal diversity, two to four isolates with different colony morphotypes per species and per volunteer were frozen to constitute the collection LaCa (Supplementary Table S3). Species identification was confirmed with full genome sequencing as detailed in the section “DNA extraction, amplification and sequencing” and “Genomic analyses”.

The clean deletion of *tyrA* in *S. aureus* Newman and USA300 JE2, as well as the restoration of this same gene in Sa A were generated via allelic exchange with the thermosensitive plasmids pIMAY:Δ*tyrA* and pIMAY:*tyrA*(T) [104, 105]. Strains, plasmids and primers used for this are provided in Supplementary Tables S7 and S8. Briefly, 500 bp flanking regions upstream or downstream of the *tyrA* gene were amplified from chromosomal DNA using A/B and C/D primers with overlapping sequences. A 1 kb deletion fragment with an ATG-TAA scar was created using spliced overlap extension PCR (SOE-PCR). Similarly, the reversion cassette to remove the frameshift in Sa A was created by removing the inserted nucleotide in primers B and C, as detailed in Supplementary Table S8. Fragments were cloned into pIMAY by restriction digestion and transformed via heat shock into *E. coli* IM08B [106]. The resulting plasmids were confirmed by Sanger sequencing and used to transform electrocompetent cells of *S. aureus* Newman and USA300 JE2 (pIMAY:Δ*tyrA)* or Sa A (pIMAY:*tyrA*(T)). For allelic exchange, a single clone per construct was resuspended in 900 µL basic medium (BM) and serially diluted before plating on BM + chloramphenicol (Cm) at 37 °C. To promote genome integration of the thermosensitive plasmid, colonies were cultured in TSB at 30 °C with shaking for several days, with daily subcultures. Plasmid excision was induced by serial dilution and plating on TSA containing 1 µg/mL anhydrotetracycline (ATc), which inhibits cells retaining pIMAY. After 2 days at 30 °C, colonies were screened on TSA and TSA + Cm. Clones that grew only on TSA were selected. Colony PCR (Phire Hot start II DNA polymerase, Thermofisher Scientific) and Sanger sequencing (Eurofins Genomics) were used to confirm the presence of the deletion or restored *tyrA* in the chromosome. In the case of Newman ∆*tyrA,* whole genome sequencing (Novogene) was followed by analysis using *breseq *[107], confirming the absence of additional genetic changes between the mutant and its respective wild-type isolate.

Plasmidic complementation of the *tyrA* deletions in the genetic backgrounds Newman and USA300 JE2 was performed as follows. The *tyrA* locus including a ~ 140 bp upstream region was amplified from genomic DNA isolated from *S. aureus* using primer pair OKB7 (Supplementary Table S8). The resulting fragment was digested after a clean-up step using BamHI and EcoRI, ligated into pRB473 [108] and subsequently transformed via heat shock into *E. coli* IM08B. The obtained plasmid was confirmed by Sanger sequencing and used to transform Newman ∆*tyrA* and USA300 JE2 ∆*tyrA* by electroporation.

### Culture conditions

Bacterial strains were routinely cultivated from frozen stocks on tryptic soy agar (TSA) supplemented with 5% sheep blood, under the oxygenation conditions appropriate for each species as detailed in Supplementary Table S9. For all experiments, bacterial precultures were then prepared from colonies and incubated under species-specific conditions. In general, aerobic species were cultivated in BHI (Roth) (*Staphylococci* spp., *Klebsiella* spp.) or BHI + 0.2% Tween 80 (Sigma) (Corynebacterium spp., *Moraxella* spp.) at 37 °C with agitation. *Cutibacterium* spp. were cultivated in TSB (Oxoid) without agitation, using Falcon tubes completely filled to minimize oxygen exposure. For strict anaerobes with limited growth in liquid broth, bacterial suspensions were prepared by harvesting colonies from fresh agar plates (Supplementary Table S9). Bacterial cells from the preculture or from the colonies were then washed twice in sterile PBS prior to concentration adjustment. Subsequent experiments were conducted in variations of Synthetic Nasal Medium (SNM, SNM3, SNM20), which were prepared from filter-sterilized stock solutions of salts, amino acids, organic acids, glucose, vitamins and trace elements as described previously [36]. These media mimic the nutrient composition of human nasal secretions, with SNM3 and SNM20 having three- or 20-fold higher concentrations of glucose, organic compounds, and amino acids, compared to the base SNM. Stock solutions were stored at 4 °C until use and pre-warmed before inoculation.

Depending on the assay, the medium was supplemented with the six amino acids missing from SNM either individually or as a mixture (100 µM each), using filter-sterilized stock solutions. These amino acids included tyrosine, methionine, glutamine, asparagine, aspartate (AppliChem) and isoleucine (Roth). In general, growth kinetics over 24 h to 36 h were conducted in 48-well plates containing 500 µL culture per well, sealed with a plastic film, and incubated at 37 °C with continuous orbital shaking. OD_600nm_ was measured every 15 min using an Epoch plate reader (BioTek). For each condition, two to three technical replicates and at least three biological replicates were performed. Blank wells were included for background subtraction.

### Binary interactions between nasal strains

Volunteers A, B, C, J, and K were selected for binary interaction experiments based on the composition of their nasal microbiota, prioritizing individuals with clearly defined *S. aureus* carriage or non-carriage status and a microbiota structure that was both diverse and representative of major CSTs. *S. aureus* carriage was defined as a positive culture from anterior nasal swabs together with the detection of *S. aureus* sequences in 16S rRNA gene amplicon data (Fig. 1). The *S. aureus* carriage status of each individual was confirmed through multiple samplings conducted either in a previous metagenomic study [43] or in parallel projects in the laboratory.

Pairwise interactions between nasal strains were assessed using the most abundant isolate of each species derived from the volunteer of interest, based on bacterial counts obtained during the strain isolation process (Supplementary methods) (Supplementary Table S3). Interaction assays were conducted as described previously [44]. Briefly, 500 µL of the suspension of a background species, or PBS as control, was spread as a lawn on SNM agar containing bromocresol blue as a stain in rectangle plates (Singer). The plate was incubated at 37 °C for 6 h under appropriate oxygenation conditions to foster growth and metabolism of the background species (Supplementary Table S9). Remaining volumes of bacterial suspensions were aliquoted into a 96-well plate and stored at 4 °C until pinning. For pinning, bacterial suspensions from the microplate were homogenized and applied manually on top of the lawn using 96-pins pads (Singer), including 8 technical replicates per pinned strain. Each pairwise interaction assay was conducted in three independent biological replicates, performed on different days. Plates were incubated again for 7 days at 37 °C in aerobiosis and imaged under standardized conditions, using a light pad (Gaomon) and a 14.1 Mpx camera (Panasonic DMC-FZ100). We developed and used the software “BactoVision” to measure the area of arising colonies. This tool ensures standardized measurement of colonies across plates, incorporating semi-automated image enhancement features and colony detection (https://github.com/mlcolab/bactovision). Primary colony measurements are available in Supplementary Table S10. Additionally, we assessed the plates for the presence of inhibition halos in the background species caused by the pinned strains. No inhibition halos were observed.

The coefficient of variation (CV) of colony size measurements across technical and biological replicates was calculated to assess the reproducibility of the assay. CVs across the five SynComs typically ranged between 5 and 15%, indicating good reproducibility of colony size measurements across technical and biological replicates (Supplementary Fig. S2A and C). The presence of a background species did not introduce additional experimental noise (Supplementary Fig. S2B and D).

Outlier colonies resulting from pinning errors or contamination were identified and removed using Prism’s standard outlier detection prior to averaging technical replicates. To evaluate the impact of background species, variations in colony size of each pinned strain were analyzed by (i) ANOVA with Dunnett’s correction comparing colony areas on PBS versus background lawns, and (ii) calculating growth fold changes as the ratio of colony area on the background lawn to that on the PBS control. Corresponding fold-changes and adjusted *P*-values from the ANOVA tests are provided in Supplementary Table S11.

Each strain pair was tested in both orientations, with each strain serving once as the background and once as the pinned species, allowing assessment of reciprocal effects. Interaction types were then defined based on the direction and reciprocity of growth changes of the pinned species, as defined in the result section. Interactions were categorized as detailed in Fig. 2B, then plotted as network diagrams.

### Effect of SynComs on *S. aureus* proliferation

Synthetic communities (SynComs) were assembled using the same isolates used in the binary interaction experiments. For SynComs B and K, the species *K. oxytoca* and *K. michiganensis* were excluded due to their overgrowth of other species and their absence in the results of the 16S rRNA gene amplicon sequencing (Supplementary Table S1). Native *S. aureus* strains were not included in the initial SynCom mix, so that the effect of a single *S. aureus* strain could be evaluated in each community in subsequent experiments. Following preculture in rich medium in appropriate conditions (Supplementary Table S9), each strain was resuspended in PBS + 30% glycerol to OD_600nm_ = 5, after which the suspensions were combined in an equal ratio. Aliquots of the resulting SynCom were stored at − 80 °C until further use. The absence of contaminants and the presence of all expected species were verified by plating on TSA + Blood plates and 16S rRNA Illumina gene amplicon sequencing (Fig. 3B), as performed on nasal swabs.

For synthetic culture assays, OD_600nm_ = 1 suspensions were prepared in PBS from the frozen SynCom and a fresh preculture of *S. aureus*. Twenty microliters of the suspensions of *S. aureus*, the SynCom or both (1:1 ratio) were evenly distributed onto SNM agar in 12-well plates and incubated at 37 °C for 3 days. The agar was then flooded with 1 mL PBS to harvest cells. One fraction of this suspension was used to determine bacterial concentrations: *S. aureus* counts from mono- and cocultures were determined on Chromagar *S. aureus* [109], while SynCom viability and growth were verified by plating SynCom monocultures on TSA + Blood agar and count total colonies (Supplementary Fig. S6C). The remaining fraction (≈ 500 µL) of the suspensions derived from SynCom monocultures and coculture with *S. aureus* was stored at − 80 °C before 16S rRNA Illumina gene amplicon sequencing, as performed on nasal swabs.

For *S. aureus* exposure to secreted metabolites, each SynCom was inoculated in liquid SNM to OD_600nm_ = 0.05, and cultivated at 37 °C with agitation for 48 h. After incubation, SynCom growth was checked using OD_600nm_ measurements and platings on TSA + Blood (Supplementary Fig. S6A and B). Cultures were then filtered to obtain cell-free supernatants, which were stored at − 80 °C until use. For *S. aureus* growth assays, supernatants were diluted 1:1 with fresh sterile SNM (final concentration: 50% spent medium). Growth kinetics of *S. aureus* in SNM, in the presence or absence of SynCom-derived supernatants, were then performed as described in the “Culture conditions” section.

### Metabolome of SynComs

SynComs were cultivated in monoculture for 3 days on SNM agar at 37 °C, using the 12-well plate format as described above. After incubation, 1 mL of 80% methanol was added to each well and incubated for 20 min before recovery. The methanol suspension was recovered without scraping the SynCom lawn, and its OD_600nm_ was measured for normalization. The methanol extract was centrifuged at 12,000 rpm for 10 min and the supernatant was stored at − 80 °C in glass vials until analysis. Targeted metabolomics was performed via isotope ratio LC–MS/MS on an Agilent TQ 6495. For chromatographic separation, an InfinityLab Poroshell 120 HILIC-Z 2.7 µm, 2.1 × 150 mm (Agilent Technologies) was used. Chromatographic parameters were set as described in Agilent application note 5994-5949. TQ settings and multiple reaction monitoring were obtained from Guder and colleagues [110]. For quantification, each sample was spiked with a ^13^C-labeled *E. coli* internal standard, in which all metabolites are ^13^C-labeled. Metabolites were identified by overlaying the ^12^C sample signal with the ^13^C internal standard signal, requiring matching retention times and peak shapes. The ^13^C signal was then used to normalize the ^12^C sample signal, providing relative metabolite concentrations while correcting for ion suppression and matrix effects. Finally, the metabolite ratios of the SynComs were normalized to their respective OD_600nm_ to account for differences in the amount of material recovered from each SynCom lawn (Supplementary Table S6).

### Cocultivation of prototrophic and auxotrophic *S. aureus* strains

The transwell system (Corning) was used to perform mono- and cocultures of *S. aureus* strains with a functional (Newman WT, USA300 JE2 WT, Sa A-*tyrA*) or an impaired (Newman Δ*tyrA,* USA300 JE2 Δ*tyrA*, Sa A WT) *tyrA* gene (Supplementary Table S7). Bacterial suspensions with an OD_600 nm_ 0.015 were prepared in SNM + 100 µM tyrosine and inoculated either into the insert (500 µL, *tyrA*^−^ strains) or in the bottom part (1.5 mL, *tyrA*^+^ strains) of a 12-well transwell plate. For monocultures, the bacterial suspension of one partner was replaced by sterile SNM + tyrosine. The plate was sealed with a plastic foil and incubated for 24 h at 37 °C with agitation. Following incubation, cultures from the inserts and bottom wells were recovered, serially diluted and plated on TSA to quantify the bacterial counts of both *tyrA*^−^ and *tyrA*^+^ strains.

### In vitro identification of tyrosine auxotrophs

A biobank was constituted with 512 *S. aureus* human strains from the collections of J. Larsen [111] (JeLa, *n* = 8), J. Rudkin (JuRu, *n* = 277), students from Munich (MuSt, *n* = 202), Universitätsklinikum Tübingen (UKT, *n* = 19), and this study (LaCa, *n* = 6). This biobank included human strains of nasal (n = 380), infectious (*n* = 124) or undefined (*n *= 8) origins. Nasal strains were obtained from the nose of healthy volunteers, whereas infectious strains were derived from non-nasal human sources and associated with a reported case of *S. aureus* infection. To validate the method, a set of control strains (including well-characterized reference strains and *tyrA* mutants) was also included in the pinning assay (Supplementary Fig. S13A, B). The complete list of test and control strains is provided in Supplementary Table S7.

A visualization of the screening procedure is depicted in Fig. 6A. Bacterial suspensions of all strains were frozen in cryoprotective medium at − 80 °C in microtiter plates until use. Using the PM1 robotic colony replicator (S&P Robotics) [112], the biobank was arranged into the 384-well microtiter format on rectangle plates (VWR) containing TSA and cultivated overnight at 37 °C for recovery. For the high-throughput detection of tyrosine dependencies, a 20×-concentrated version of the SNM (SNM20) [36] was used to ensure robust growth and subsequent colony detection. The biobank was transferred on SNM20 agar either unmodified or supplemented with tyrosine alone (TYR, 100 µM) or a mixture of the six amino acids missing from SNM (6AA, 100 µM each), as well as on TSA for growth control. The screening was performed in three biological replicates, each with at least two technical replicates, resulting in a total of 757 pinned colonies. After 2 days of incubation at 37 °C, plates were imaged using a semi-automatic imager (spImager, S&P Robotics). Colony size was measured using IRIS [113] in the default mode “Colony growth”, which was also used as a growth metric. Data quality and reproducibility were assessed by visual inspection of the IRIS output and replicate correlation. To account for enhanced growth of colonies in the outer frame of each plate, colony growth was normalized based on the average increase observed in outer frame colonies.

Colony size comparisons were then conducted to assess the effects of amino acid supplementation, as outlined in Supplementary Fig. S13C. Briefly, strains forming colonies of equal or smaller size than the genetic tyrosine auxotrophs (*S. aureus* LS1 [114], *tyrA* mutants of USA300 JE2 and Newman) on SNM were classified as having a growth defect. Strains that formed larger colonies on SNM + TYR or SNM + 6AA compared to SNM were identified as stimulated by the corresponding amino acid(s). Strains that exhibited similar growth across all three tested conditions were considered unaffected by amino acid supplementation.

### Nasal colonization of gnotobiotic mice

For in vivo experiments of nasal colonization, bacterial inocula were prepared from fresh precultures of *S. aureus* or from frozen SynCom stocks. Suspensions containing either *S. aureus* alone or a mixture of *S. aureus* and the SynCom were adjusted to the desired CFU in PBS + 30% glycerol and stored at − 80 °C until use. Groups of gnotobiotic C57BL/6N mice (4–9 animals per group; 7–14 weeks old; 18–26 g; mixed sex depending on breeding) were used, with 1 to 5 mice per cage depending on group composition and sex. Mice were anesthetized by isoflurane inhalation, and 5 to 10 µL of the bacterial inoculum was applied dropwise with a pipette onto both nostrils under spontaneous breathing, with a target dose of 2 × 10^4^ CFU of *S.* *aureus* and 3 × 10^5^ CFU of the SynCom per nose. Seven days after inoculation, mice were euthanized by CO_2_ asphyxiation following surgical harvesting of nose, feces, lung and liver. All samples were vortexed for 1 min in 1 mL sterile PBS, serially diluted and plated on TSA + Blood or Chromagar *S. aureus* plates to evaluate *S. aureus* colonization (nose, feces) and the absence of systemic dissemination (lung, liver). All animal experiments were conducted in accordance with German laws after approval (IMIT 02/21G) by the local authorities (Regierungspraesidium Tuebingen).

### DNA extraction, amplification, and sequencing

Detailed procedures for DNA extractions, library barcoding, and sequencing are provided in the Supplementary methods. Briefly, for 16S rRNA gene amplicon sequencing, total microbial DNA was extracted from the sample (1 mL Amies medium in which nasal swabs were suspended, or 500 µL of bacterial suspension derived from SynCom cultures) using the HostZero Microbial DNA kit (Zymo Research), including the host DNA depletion step for nasal swab samples. DNA amplification, amplicon library pooling, and V1–V3 16S rRNA gene amplicon sequencing were conducted by the NGS Competence Center Tübingen (NCCT), as described previously [43, 115]. The 16S rRNA gene amplicon sequencing results are provided in Supplementary Tables S1 and S4, and the raw data will be made publicly available on NCBI upon publication. The DNA extraction and the whole-genome sequencing (WGS) of individual strains were performed by the NCCT or by the Center for Biotechnology Bielefeld (CeBiTec), using Illumina NovaSeq and/or PromethION devices depending on samples (Supplementary Table S3). Circular genomes are available on the BioProjects PRJNA1199961 (newly identified *Corynebacterium* species) and PRJNA1247435 (other species).

### Genomic analyses

16S rRNA gene amplicon sequencing data were analyzed as described previously [43]. For individual strains, sequencing reads were assembled into circular genomes using Flye [116], Canu [117] or Unicycler [118] depending on the sample (Supplementary methods). These genomes were then classified using pubMLST (https://pubmlst.org/), which compares MLST profiles of the sample to a proprietary database, and using the Type Strain Genome Server TYGS [41] (https://tygs.dsmz.de), which calculates digital DNA-DNA hybridization (dDDH) values between two genomes and compares them. Genome pairs with a dDDH value over 99.5% were considered of clonal origin. Genome annotation was performed using Bakta [119] (https://github.com/oschwengers/bakta) or Prokka [120] (https://github.com/tseemann/prokka).

WGS information of SynCom strains were subjected to antiSMASH (V7.0) analysis using the available web tool [121]. Obtained summary information of each isolate was compiled using a custom R script. Biosynthetic gene clusters (BGCs) were classified by the provided “Similarity confidence” feature. Additionally, we curated the obtained list of BGC types manually for BGCs with documented antimicrobial properties consulting the existing literature. Results of (antimicrobial) BGCs are shown in Supplementary Table S5.

The frequency of frameshift mutations in the *tyrA* gene across *S. aureus* isolates was determined as follows. *S. aureus* WGS data was retrieved from Refseq (*n* = 9340). The DNA sequence of *tyrA* from *S. aureu*s Newman (NWMN_1277) was used as a query in a BLAST [122] search against obtained WGS data featuring the assembly level “Chromosome”, “Complete Genome” or “Contig”. Identified loci were aligned against the query sequence using Clustal Omega provided in the msa R package [123] and the TyrA ORF was determined. Truncated versions of TyrA featuring less than 363 amino acids were classified as disrupted.

### Statistical analyses

Biological replicates were performed using independent bacterial cultures from separate overnight precultures. Most replicates were conducted on different days to capture day-to-day variation. Statistical analyses were performed using GraphPad (v9.3.1 and v10.2.1) or the R stats package. Data normality was assessed using the Shapiro–Wilk test. Differences between group means were assessed using Analysis of Variance (ANOVA) followed by Dunnett’s, Tukey’s or Šídák’s post-test comparisons, as specified in the corresponding figures. For the high-throughput screening of tyrosine auxotrophs, differences between mean colony sizes were evaluated using a *t*-test followed by Bonferroni correction to control for multiple comparisons. The medians of bacterial concentrations recovered from mice noses and feces were compared using the Kruskal–Wallis test with Dunn’s correction or Welch’s *t*-tests with Holm–Šidák correction. Adjusted *P* values below 0.05 were considered significant and displayed as *P_adj_ < 0.05, **P_adj_ < 0.01, ***P_adj_ < 0.001, ****P_adj_ < 0.0001.

## Supplementary Information

Supplementary Material 1. 

Supplementary Material 2. 

## Data Availability

Circular genomes sequenced in this study are available on NCBI (BioProjects PRJNA1199961 and PRJNA1247435). All other datasets are provided in the main and supplementary documents.
