# Supplementary material for: Amino acid availability influences Staphylococcus aureus interaction with the nasal microbiota in a strain- and community-specific manner
Source: Microbiome. 2026 Oct 23;14:237. doi: 10.1186/s40168-026-02563-x (PMC13692985; doi:10.1186/s40168-026-02563-x)
Supplement: Supplementary file 1 [file 40168_2026_2563_MOESM1_ESM.pdf]

Amino acid availability influences *Staphylococcus aureus* interaction with the nasal microbiota in a strain- and community-specific manner

Laura Camus[1,2,3,*], Jessica Franz[1], David Gerlach[4], Anna Lange[1,2], Jeffrey John Power[1,2], Marcelo Navarro- Díaz[1,2,5], Jens Stahl[1], Bernhard Krismer[1], Johanna Rapp[1,2,5], Kevser Bilici[1,2,4] Matthias Otto[6], Levin Joe Klages[6], Vladimir Starostin[7], Soyoung Ham[2,8], Jörn Kalinowski[6], Largus T. Angenent[2,8], Hannes Link[1,2,5], Simon Heilbronner[1,2,3,4,9,*]

Affiliations

1. Interfaculty Institute of Microbiology and Infection Medicine, Department of Infection Biology, University of Tübingen, 72076 Tübingen, Germany.
2. Cluster of Excellence EXC 2124 Controlling Microbes to Fight Infections, 72076 Tübingen, Germany.
3. German Centre for Infection Research (DZIF), Partner Site Tübingen, Tübingen, Germany.
4. Faculty of Biology, Microbiology, Ludwig-Maximilians-University of Munich, 82152 München, Germany.
5. M3 Research Center, Otfried-Müller-Straße 37, University of Tübingen, 72076, Tübingen, Germany.
6. Center for Biotechnology (CeBiTec), Bielefeld University, 33615 Bielefeld, Germany.
7. Cluster of Excellence Machine Learning: New Perspectives for Science, University of Tübingen, Maria-von-Linden-Straße 6, 72076, Tübingen, Germany.
8. Environmental Biotechnology Group, Department of Geosciences, University of Tübingen, 72076, Tübingen, Germany.
9. Lead contact, Simon Heilbronner (Simon.Heilbronner@bio.lmu.de)

*** Corresponding authors**
Laura Camus, lauracamus.bio@gmail.com
Simon Heilbronner, Simon.Heilbronner@bio.lmu.de

Supplementary information

Supplementary methods – Pages 2 to 4.
Supplementary figures – Pages 5 to 20.
Supplementary tables – Separated Excel file. Table list and legends on page 21.
Supplementary references – Page 23.

Supplementary methods

Isolation and characterization of nasal communities

The volunteers A, B, D, E, F, G, H, I, and J had previously participated in a metagenomic study conducted in our laboratory[1], while the volunteers C and K were newly recruited. In all cases, participants were selected for the current study based on the following criteria: (1) a healthy status for at least two weeks prior to sampling, with no known illness or antibiotic treatment that could have influenced the nasal microbiota or immune status; (2) availability for daily sampling over three consecutive days; and (3) inclusion of both male and female participants, although full gender parity could not be achieved. Nasal samples were collected using sterile flocked swabs (eSwab, Copan) from both anterior nares of each volunteer. Swabs were immediately suspended in 1 mL Amies transport medium, stored at 4°C, and processed within 2 h of collection. For each volunteer, a total of seven nasal swabs were collected over three consecutive days to conduct metagenomic sequencing (six swabs) and strain isolation (one swab).

Metagenomic sequencing. Six swabs were individually subjected to metagenomic DNA extraction right after sampling, using the HostZero Microbial DNA kit (Zymo Research) including the host DNA depletion step. The resulting DNA was pooled prior to V1-V3 16S rRNA gene metagenomic sequencing as described elsewhere[1,2]. For this, the genomic DNA was quantified with a Qubit dsDNA BR/HS Assay Kit (Thermo Fisher) and normalized to a maximum of 5 ng Input for library preparation. The first step PCR was performed in 25 µl reactions including KAPA HiFi HotStart ReadyMix (Roche), custom primers and template DNA (PCR program: 95°C for 3 min, 30x (98°C for 20 sec, 69°C for 15 sec, 72° for 15 sec), 72°C for 5 min). First PCR products were purified using 28µl AMPure XP beads and eluted in 50µL 10mM Tris-HCl. Indexing was performed in the second step PCR including KAPA HiFi HotStart ReadyMix (Roche), index primer mix (Illumina Nextera XT Index Kit v2), purified first PCR product as template (PCR program: 95°C for 3 min, 8x (95°C for 30 sec, 55°C for 30 sec, 72°C for 30 sec), 72°C for 5 min). After another bead purification (20µl AMPure XP beads, eluted in 30µL 10mM Tris-HCl) the libraries were checked for correct fragment length on an agarose gel, quantified with a Qubit dsDNA BR Assay Kit (Thermo Fisher) and pooled equimolarly. The pool was sequenced on an Illumina MiSeq device with a v2 sequencing kit with 101,8,8,401 read length.

Strain isolation and first identification. The seventh nasal swab was subjected to strain isolation procedure as described previously[3]. A first fraction of the nasal swab was serially

diluted (10^{-1} to 10^{-3}) and plated on four different rich agar media: Columbia agar with 5% sheep blood (Fisher Scientific), Schaedler agar with 5% sheep blood, Chocolate agar with 10% sheep blood, Schaedler agar supplied with 5% horse blood, kanamycin, and vancomycin (Universitätklinikum Tübingen). Plates were incubated for 3 to 10 days under aerobic, anaerobic (Anaerocult® A, Merck) or CO_2-enriched (21% O_2, 5% CO_2) conditions at 37°C. In parallel, the second fraction of the swab was used to inoculate two liquid broths: BHI (Roth) supplemented with 0.2% Tween 80 (Sigma) in agitated glass flasks for aerobic growth at 37°C during 12 h, and Thioglycollate medium (Sigma) in static canonical tubes for anaerobic growth at 37°C during 48 h. The resulting cultures were then serially diluted (10^{-5} to 10^{-7}) and plated on Columbia agar with 5% sheep blood or on Schaedler agar with 5% sheep blood and incubated for 2 to 7 days in aerobiosis or anaerobiosis. For each agar plate, the colony morphotypes were differentiated by visual inspection at the end of the incubation period, and the bacterial concentration of each morphotype was determined by colony enumeration. Two colonies per morphotype were picked and reisolated on the appropriate agar medium prior to species identification using matrix-assisted laser desorption/ionization-time-of-flight mass spectrometry (MALDI-TOF MS) as described elsewhere[4]. To promote cell lysis and accurate species identification, 1µL of 70% formic acid was added to the smeared colonies on the MS target plate prior addition of the MS matrix. The analysis was then performed by the Institute of Medical Microbiology and Hygiene (University Hospital Tübingen, Germany) using the Bruker Daltonik MALDI Biotyper and a composite database of 9128 Main Spectrum Profiles (Bruker Daltonik Database, Filamentous Fungi, Clinical SR database, Database Tübingen). Species identification was considered explicit when the MS score exceeded or was equal to two. Two to four isolates per species and per volunteer were stored to constitute the LaCa collection.

Whole genome sequencing of individual isolates

Nanopore sequencing. Nanopore sequencing was performed by the Center for Biotechnology (CeBiTec Bielefeld) on a subset of strains **(Table S3)**. Genomic DNA was extracted from cell pellets using the Macherey-Nagel NucleoSpin Microbial DNA Kit. Sequencing libraries were prepared with the Native Barcoding Kit (SQK-NBD114.96, Oxford Nanopore). The DNA repair and end-prep steps were performed with the recommended reagent volumes for samples with DNA concentrations between 8 ng/µL and 18 ng/µL. For samples with DNA concentrations below 8 ng/µL, the reagent volumes were doubled, and for samples with concentrations above 18 ng/µL, the volumes were halved. Barcoding was performed using equal amounts of end-prepped DNA, blunt/TA ligase mix, and EDTA.

Sequencing was carried out using FLO-PRO114M flow cells on a PromethION P2 Solo device. Base calling was performed with Guppy base caller v6.3.9.

Hybrid sequencing. Hybrid sequencing was conducted by the NGS Competence Center Tübingen (NCCT) on a subset of strains **(Table S3)**. For bacterial lysis, a cell pellet was resuspended in 600 µL ATL buffer (Qiagen #939011) and transferred to a ZR BashingBead Lysis Tube (Zymo Research #S6012-50). The tube was vortexed horizontally for two minutes on a vortex shaker. To optimize the DNA extraction, the supernatant was taken off and digested with RNAse A (Qiagen). The DNA was then automatically purified with the QIAamp 96 QIAcube HT kit (Qiagen #51331) with additional proteinase K on a QIAcube HT following the manufacturers' instructions. The genomic DNA was quantified with a Qubit dsDNA BR Assay Kit (Thermo Fisher) and DNA integrity was checked by agarose gel electrophoresis. Libraries for short-read sequencing were prepared using the Illumina DNA Prep (M) Tagmentation kit according to the manufacturer's protocol with 500 ng DNA input and 5 cycles indexing PCR. Libraries were checked for correct fragment length on an Agilent 2100 BioAnalyzer and pooled equimolarly. The pool was sequenced on an Illumina NovaSeq device with a NovaSeq 6000 SP v1 sequencing kit with 2 x 150 bp read length. The sequencing was demultiplexed with bcl2fastq (v2.19.0.316) and quality checked with fastq (v0.20.1) and visualized with MultiQC (v1.7). For long-read sequencing the Ligation Sequencing Kit (LSK) 109 (Oxford Nanopore) was used with native barcoding, following the manufacturer's protocol, with 500 ng DNA input per sample. Library size was assessed on a FEMTO Pulse (Agilent) and libraries were pooled equimolarly before sequencing on a FLO-PRO002 flow cell on a Nanopore PromethION device. Base calling was performed in high-accuracy mode with Guppy base caller v4.0.11 and with MinKNOW operating software v20.06.18.

Genome assembly and annotation. Long read sequencing reads were assembled into circular genomes using Flye[5] and annotated with Bakta[6]. Hybrid sequencing were assembled using the nf-core pipeline bacass (https://nf-co.re/bacass/2.0.0/), which includes Unicycler[7] and Canu[8] for assembly into circular genomes and Prokka[9] for annotation.

Supplementary figures

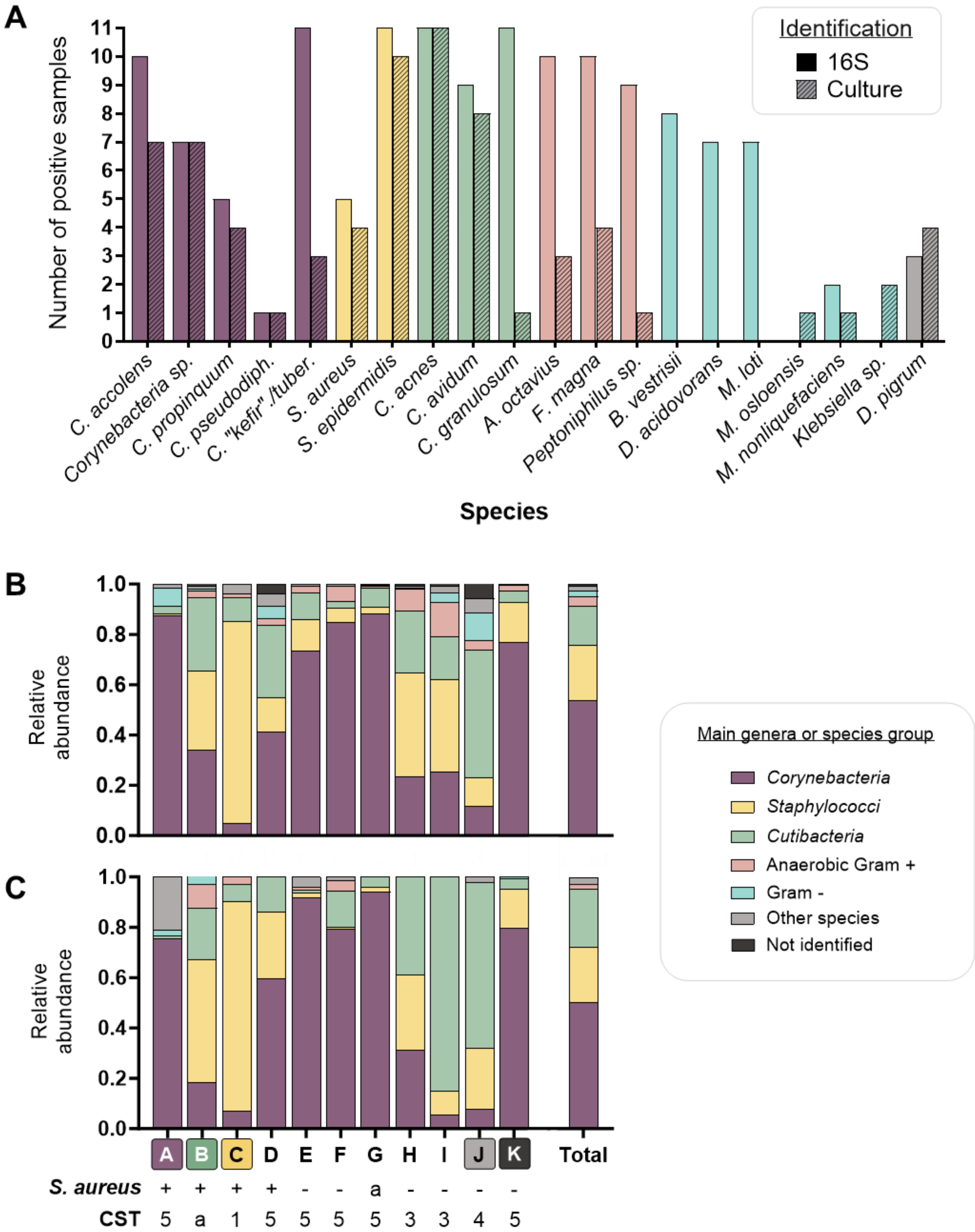

Fig. S1: **Comparison of 16S rRNA gene sequencing and culturomics approaches.**
A. Bar plot depicting the number of samples in which each species was detected using 16S rRNA gene sequencing (solid bars) or culturomics (hatched bars), across 11 volunteers. Colors correspond to the main genera or species groups shown in panels B and C.

B, C. Relative species abundance determined by 16S rRNA gene sequencing of nasal metagenomic DNA (**B**) or by isolation and enumeration of nasal species on plates (**C**), from the nasal microbiomes of eleven healthy volunteers (A-K). Each color designates the genus or the group of the 20 most abundant species across all volunteers. Less abundant species were assigned to “Other species”. Species with a missing or unclear identification were assigned to “Not identified”. Below the bars, “+” indicates the volunteers in which *S. aureus* was detected by both techniques, and numbers correspond to the community state type (CST) assigned to the corresponding community. “a” refers to ambiguous *S. aureus* carriage (detected by only one method) or CST classification (balanced proportion of several species). Each bar displays the results of one replicate.

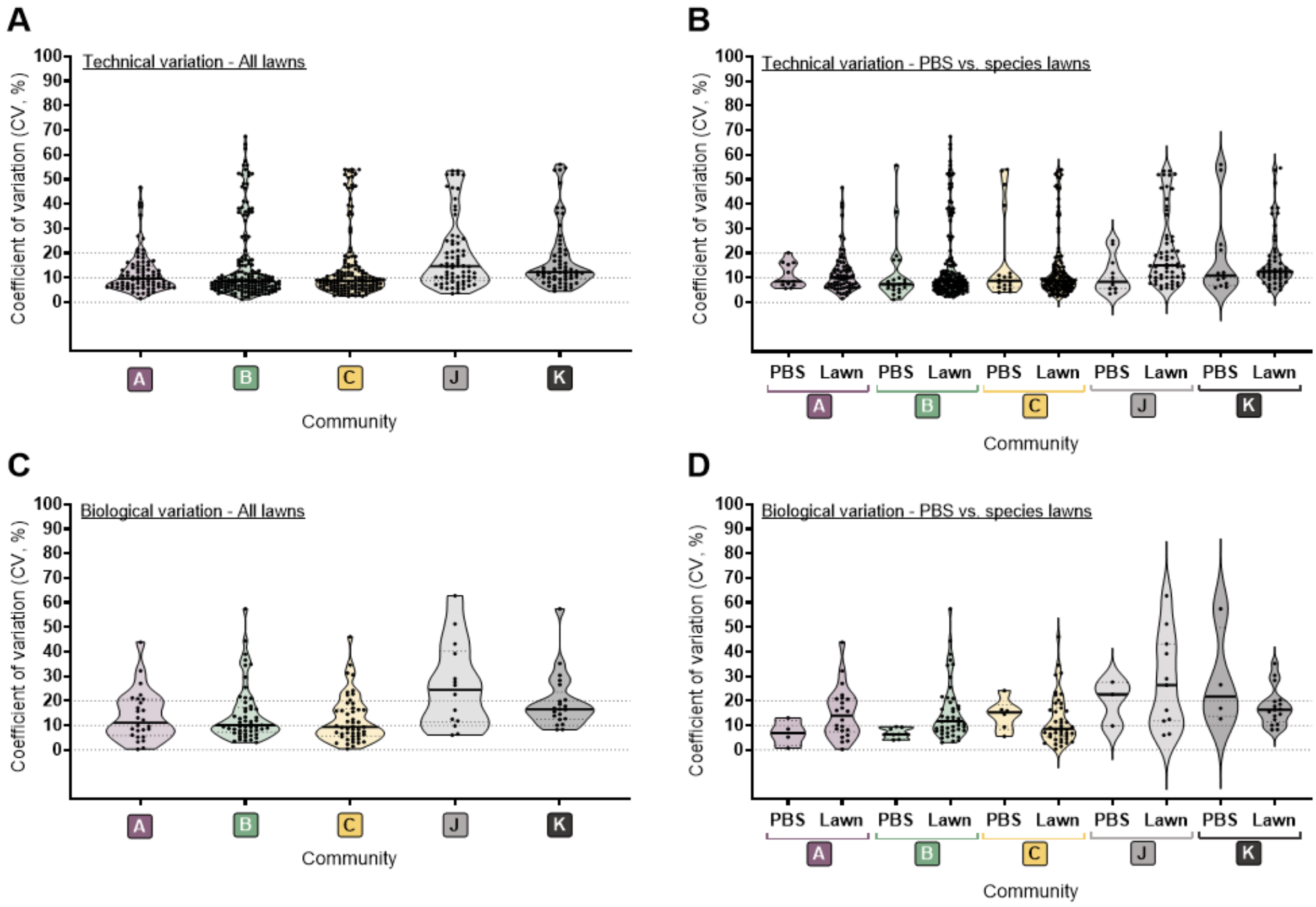

Figure S2: Technical and biological variability in colony size measurements across communities and lawn conditions.

A, B. Coefficient of variation (CV, %) of colony area measurements obtained across technical replicates for five SynComs (A, B, C, J, K). For each pinned species within a community, colony areas from the 8 technical replicates (pins) were used to calculate a CV across these 8 measurements, for each biological replicate. Each dot represents one biological replicate. CVs are shown either pooled across all lawns (**A**) or separated by lawn condition, comparing colonies pinned on the PBS control versus colonies pinned on a species lawn (**B**).

C, D. Coefficient of variation (CV, %) of colony area measurements obtained across biological replicates for five SynComs (A, B, C, J, K). For each pinned species within a community, colony areas were first averaged across the 8 technical replicates to yield a mean colony size per biological replicate. The CV was then calculated across the 3 biological replicates. Each dot represents one experiment. CVs are shown either pooled across all lawns (**C**) or separated by lawn condition, comparing colonies pinned on the PBS control versus colonies pinned on a species lawn (**D**).

For all panels. Horizontal solid lines indicate median CV values, and dotted lines mark the 0%, 10 % and 20 % CV thresholds.

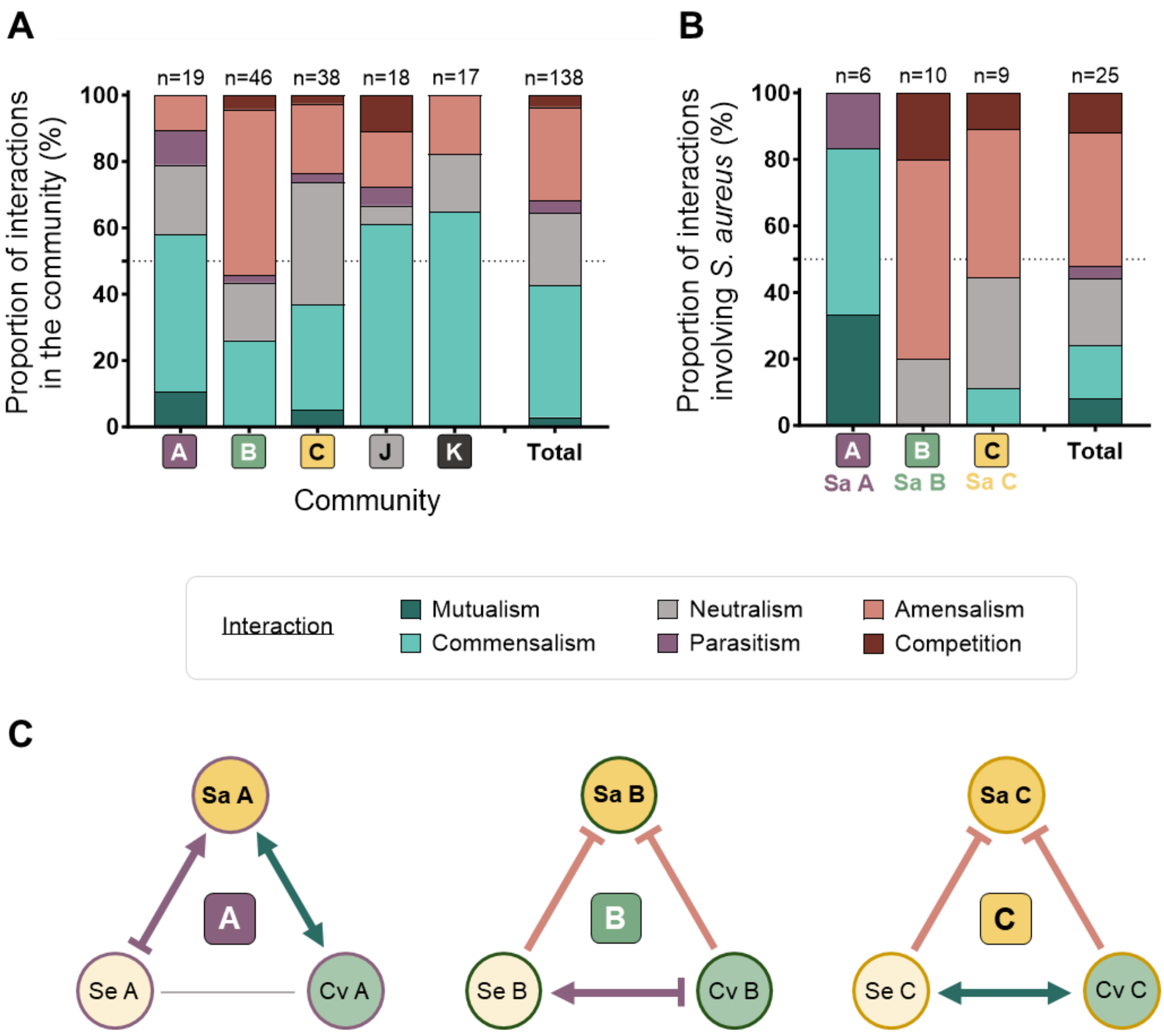

Fig. S3: Binary interactions in the nasal communities A, B, C, J and K.
For each community, interactions were determined for every pair of nasal strains on SNM agar and using colony area as a growth metric. Changes in the colony area were used to define the type of interaction. Primary colony measurements, growth fold-changes and statistical results are detailed in Tables S10 and S11.

A, B. The interactions between all strains (**A**) or involving *S. aureus* (**B**) were counted to plot their proportion within each community. The total number of interactions determined within each community is marked on the top of the bar.

C. Binary interactions between the strains of *S. aureus* (Sa), *S. epidermidis* (Se) and *C. avidum* (Cv) isolated from the communities A, B and C. Interactions were categorized as in the panels A and B.

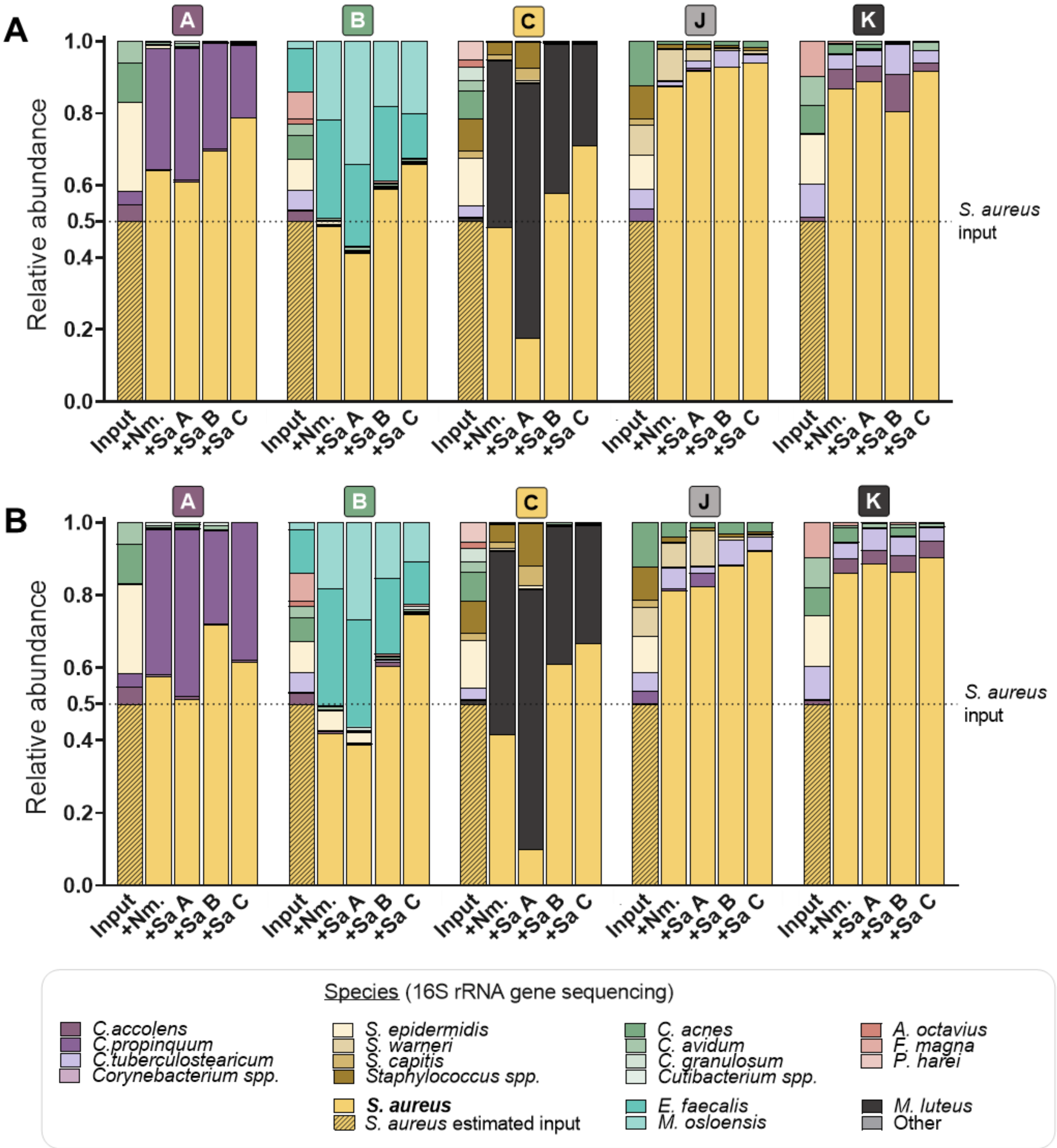

Fig. S4: *S. aureus* proliferation in mono- and co-cultivation with Syncoms.
Relative species abundance determined by 16S rRNA gene sequencing from the estimated input (frozen SynCom + *S. aureus* mixed to a 1:1 ratio) or SynComs cultivated 3 days on SNM agar with the *S. aureus* strains Newman (Nm.), Sa A, Sa B or Sa C. Each color designates a species. Less abundant species were assigned to "Other species". Each bar displays the results of the biological replicates R1 (**A**) or R2 (**B**), except for the conditions "Input" which were sequenced only once. Complete data is detailed in Table S4.

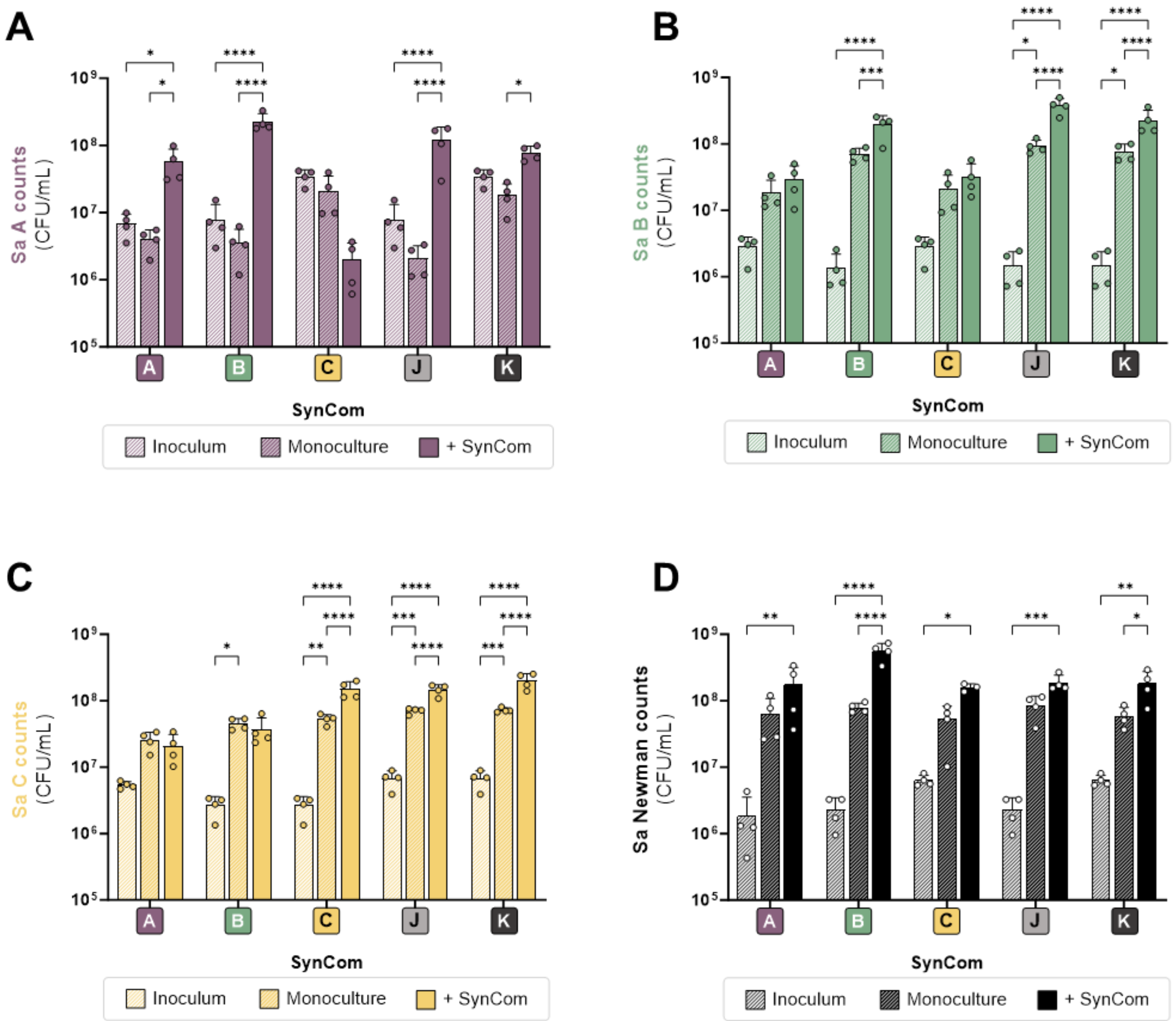

Fig. S5: *S. aureus* proliferation in mono- and co-cultivation with Syncoms.
A-D. Barplots depicting the bacterial counts of *S. aureus* in the inoculum, and after incubation for 3 days in monoculture and in synthetic culture with five different SynComs (A, B, C, J, K) on SNM agar. Each bar displays the mean + SD of four biological replicates. Statistical significance is indicated by $*P_{adj}<0.05$, $**P_{adj}<0.01$, $***P_{adj}<0.001$, $****P_{adj}<0.0001$ (Two-way ANOVA with Tukey's correction). Panels display the results for Sa A (**A**), Sa B (**B**), Sa C (**C**) and Sa Newman (**D**).

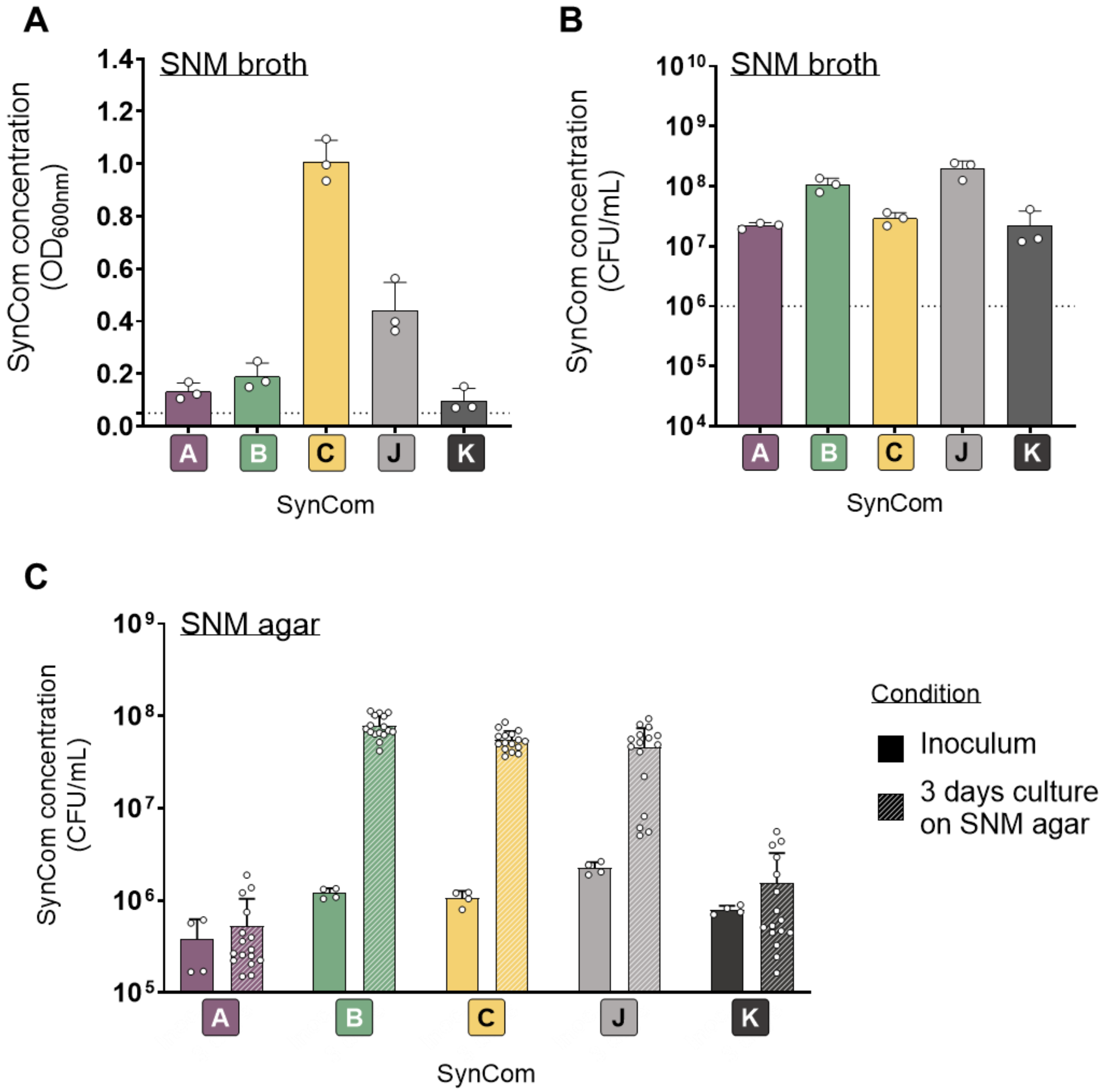

Fig. S6: Growth of the SynComs A, B, C, J and K in SNM.

A, B. Barplots showing the bacterial growth of five SynComs after 48h of culture in SNM broth, prior to filtration to generate the supernatants used in Fig. S6. Growth was measured by OD600nm (**A**) or enumeration on plates (**B**). Dotted lines indicate the initial OD_{600nm} or estimated CFU inoculum. Each bar displays the mean + SD of three biological replicates.

C. Barplots showing the bacterial growth of five SynComs in the inoculum, and after incubation for 3 days in monoculture on SNM agar, from the experiments shown in Fig. 3B and C. Each bar displays the mean + SD of four (inoculum) or sixteen (SynCom monoculture) biological replicates.

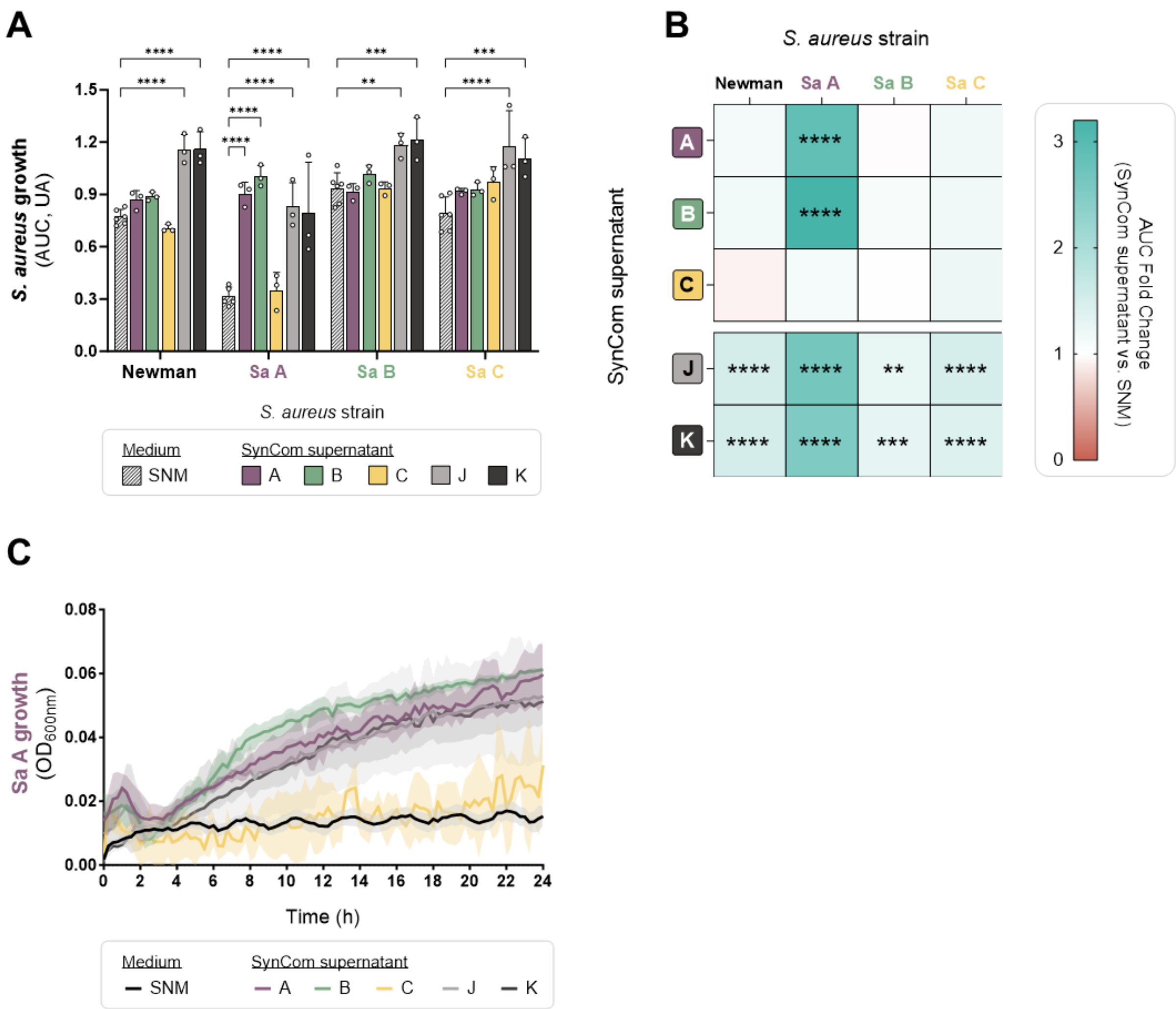

Fig. S7: Impact of SynCom supernatant on *S. aureus* proliferation in SNM.

A. Barplots showing the bacterial growth of four *S. aureus* strains cultivated in SNM or in the presence of 50% concentrated spent culture supernatant of the SynComs A, B, C, J and K. Each bar displays the mean AUC + SD from 24 h growth kinetics.

B. Heatmap showing the AUC fold-change induced by the supernatant of the five SynComs in SNM, for four *S. aureus* strains. Positive fold-changes (verdigris) and negative fold-changes (red) indicate increases or decreases in *S. aureus* growth with the supernatant relative to the SNM control, respectively.

C. Complete growth kinetic of Sa A in SNM or in the presence of the supernatant of the five SynComs. Lines show the mean ± SD of OD_{600nm} measurements, taken every 15 minutes over 24 hours.

For all panels: The data is based on at least three biological replicates of OD_{600nm} growth kinetics. Statistical significance is indicated by $*P_{adj}<0.05$, $**P_{adj}<0.01$, $***P_{adj}<0.001$, $****P_{adj}<0.0001$ (Two-way ANOVA with Dunnett's correction).

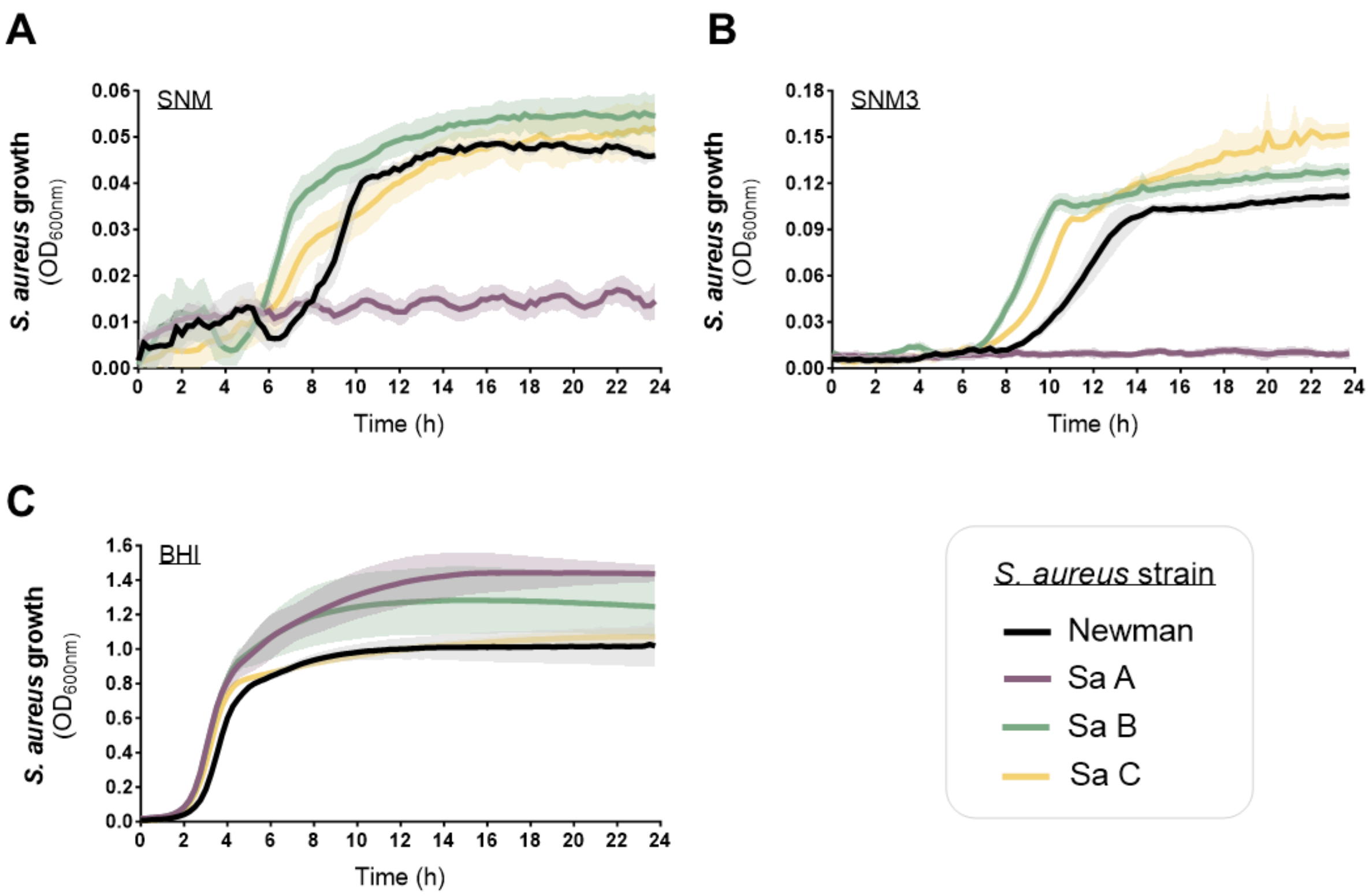

Fig. S8: Proliferation of four *S. aureus* strains in various media.
Growth kinetics in SNM (**A**), SNM3 (**B**) or BHI (**C**) for the *S. aureus* strains Newman, Sa A, Sa B or Sa C. Lines represent the mean ± SD of at least three biological replicates of OD_{600nm} measurements, taken every 15 minutes over 24 hours.

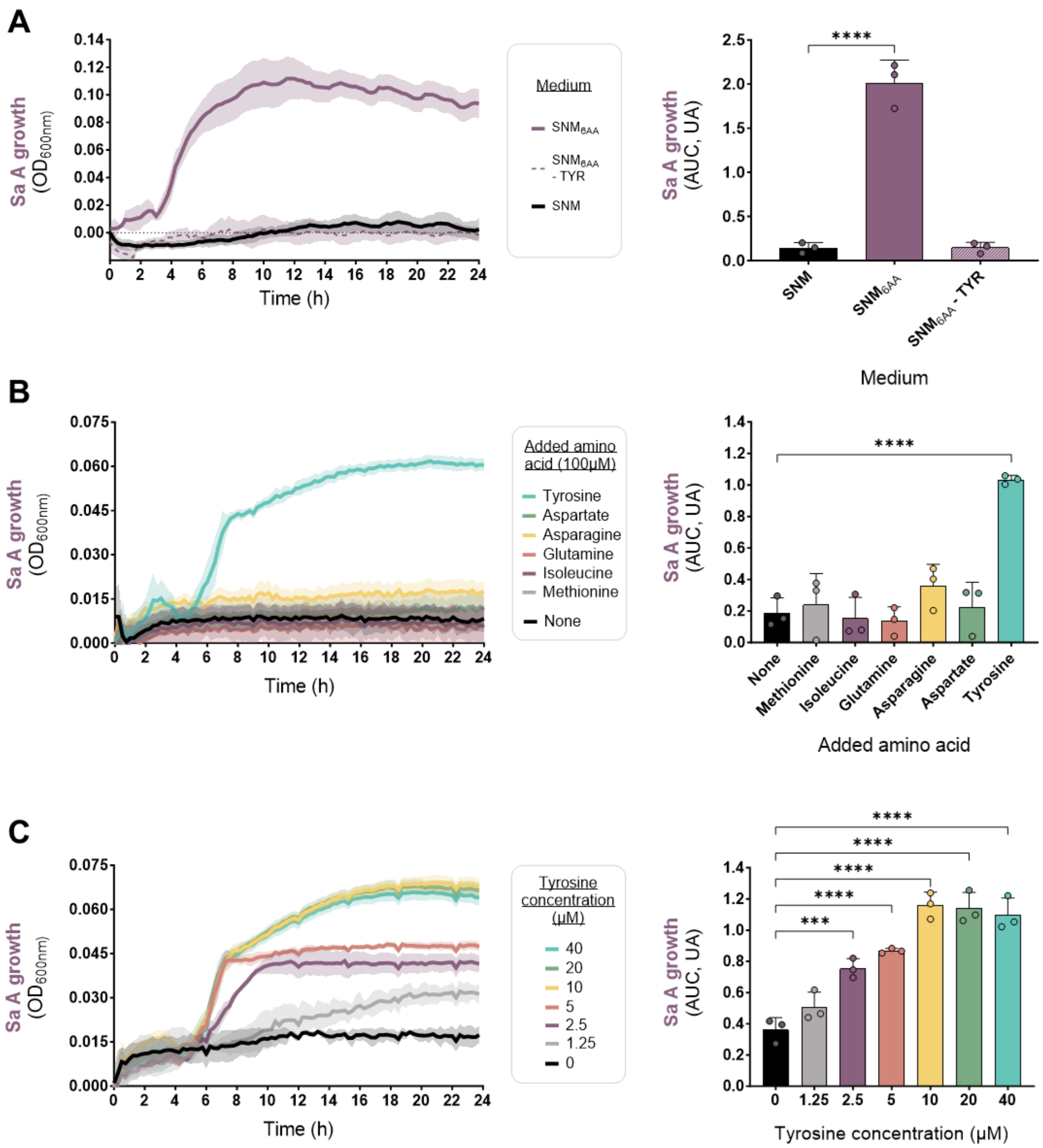

Fig. S9: **Impact of amino acid supply on the proliferation of *S. aureus* Sa A in SNM.**

For all panels: growth kinetics are displayed on the left and corresponding AUC bar plots are shown on the right. Lines or bars represent the mean ± SD of three biological replicates of OD_{600nm}, taken every 15 min over 24 hours. Statistical significance is indicated by $^{***}P_{adj}<0.001$, $^{****}P_{adj}<0.0001$ (ANOVA with Dunnett's correction).

A. Proliferation of Sa A in SNM, in SNM supplemented with a mixture of the six amino acids missing from SNM (6AA), or with the same mixture from which tyrosine was removed (6AA-TYR). 100 µM of each amino acid were used.

B. Proliferation of Sa A in SNM, or in SNM supplemented with 100µM of each individual amino acid missing from SNM.

C. Proliferation of Sa A in SNM, or in SNM supplemented with increasing concentrations of tyrosine.

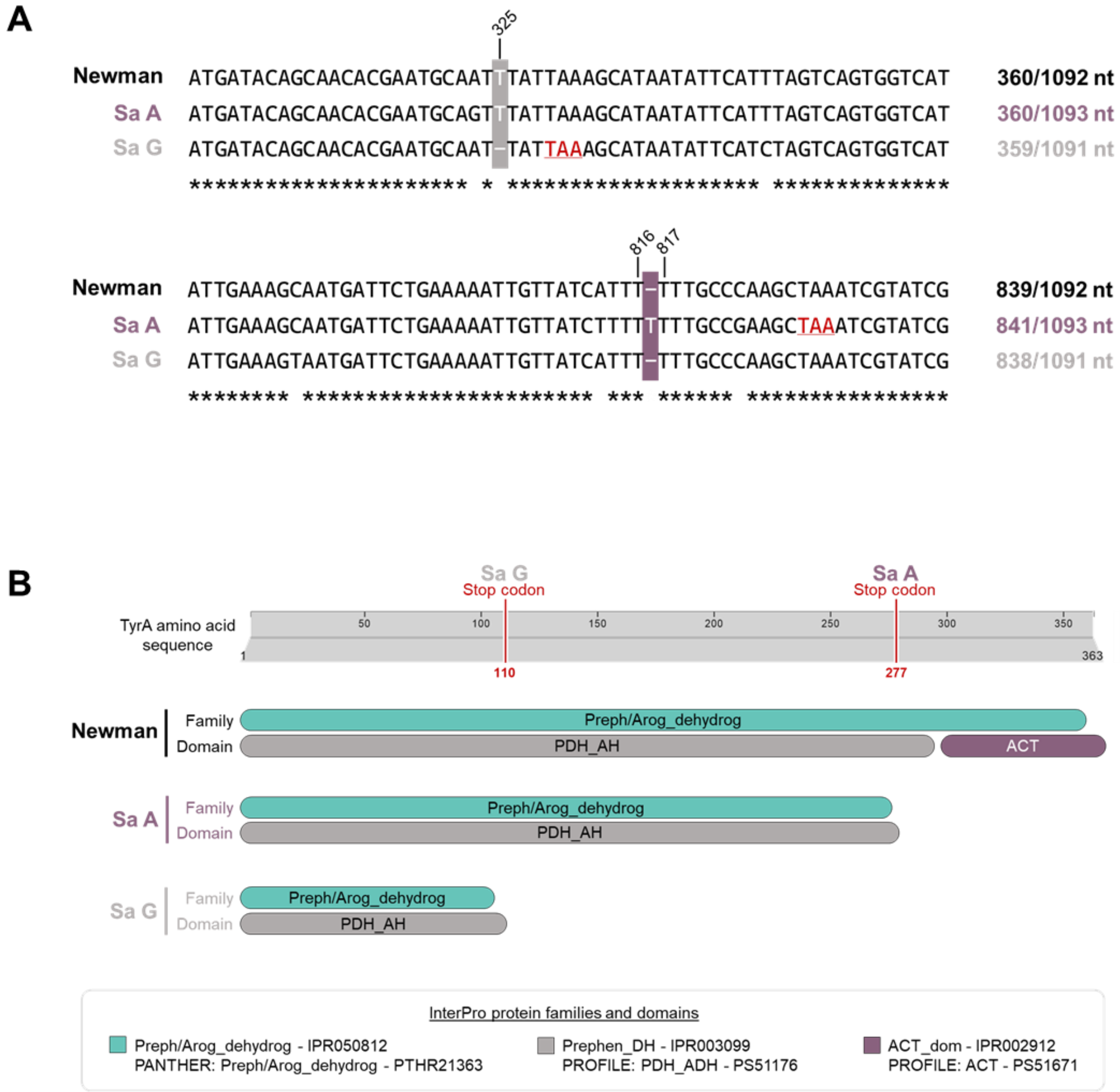

Fig. S10: Identification of deleterious mutations in the *tyrA* gene of Sa A and Sa G.

A. Sequence alignment of the *tyrA* gene *S. aureus* Newman, Sa A and Sa G. The T insertion and deletion events are highlighted in purple and gray, respectively, with the resulting stop-codon marked in red. Nucleotide positions of these mutations relative to Newman sequence are shown at the top. Adapted from Clustal Omega multiple sequence alignments.

B. Protein family and domain prediction of TyrA from *S. aureus* Newman, Sa A and Sa G. The top scale shows the full amino acid sequence of TyrA and highlights the positions of the stop codons identified in Sa A and Sa G. Adapted from InterPro. PDH_AH: prephenate/arogenate dehydrogenase. ACT: domain found in aspartate kinase, chorismate mutase and TyrA.

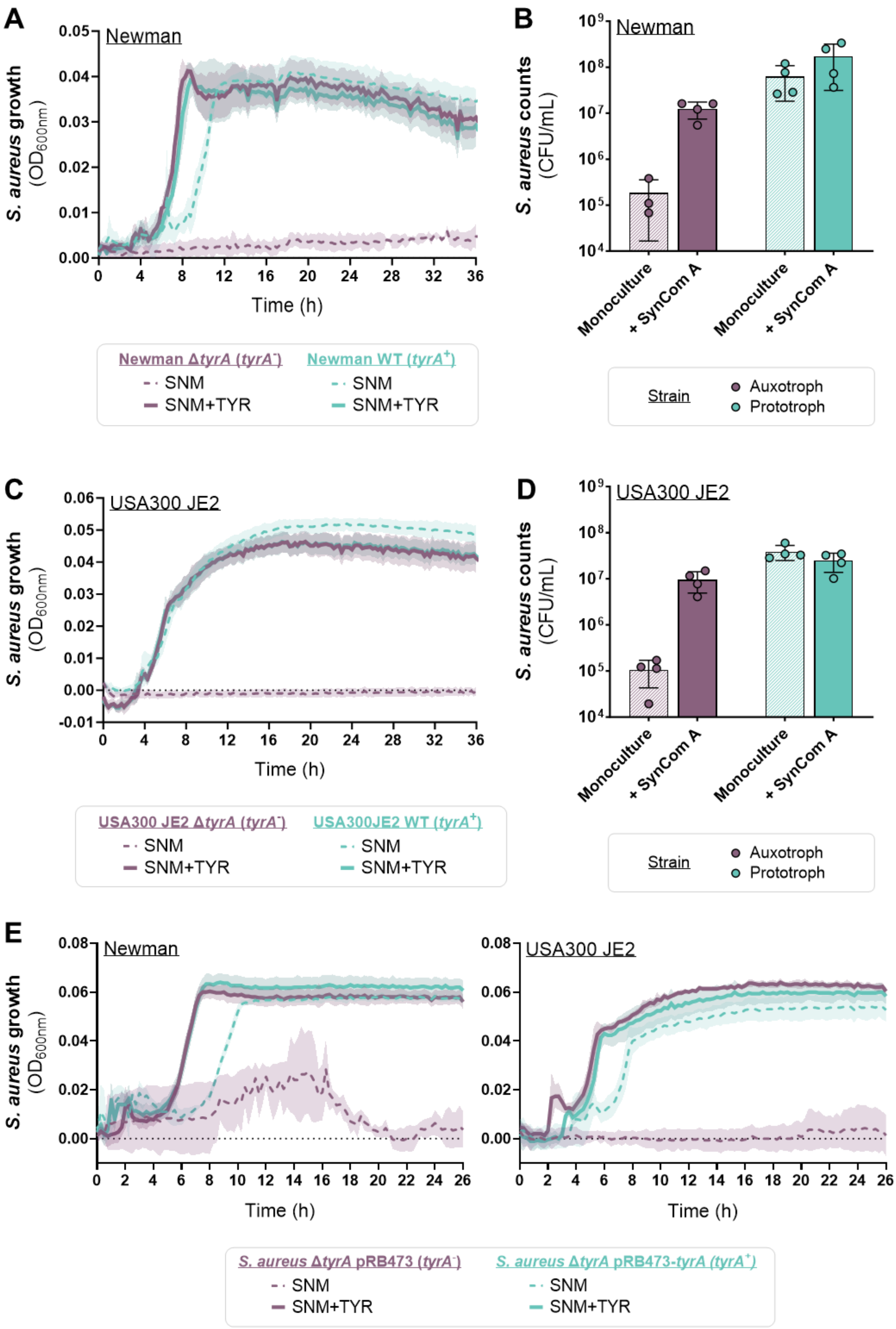
A
Newman
S. aureus growth (OD600nm)
Time (h)
Newman ΔtyrA (tyrA⁻)
SNM
SNM+TYR
Newman WT (tyrA⁺)
SNM
SNM+TYR
B
Newman
S. aureus counts (CFU/mL)
Monoculture
+ SynCom A
Monoculture
+ SynCom A
Strain
Auxotroph
Prototroph
C
USA300 JE2
S. aureus growth (OD600nm)
Time (h)
USA300 JE2 ΔtyrA (tyrA⁻)
SNM
SNM+TYR
USA300JE2 WT (tyrA⁺)
SNM
SNM+TYR
D
USA300 JE2
S. aureus counts (CFU/mL)
Monoculture
+ SynCom A
Monoculture
+ SynCom A
Strain
Auxotroph
Prototroph
E
Newman
USA300 JE2
S. aureus growth (OD600nm)
Time (h)
Time (h)
S. aureus ΔtyrA pRB473 (tyrA⁻)
SNM
SNM+TYR
S. aureus ΔtyrA pRB473-tyrA (tyrA⁺)
SNM
SNM+TYR

Fig. S11: Effect of *tyrA* deletion and complementation on the proliferation of *S. aureus* Newman and USA300 JE2 in SNM.

For all panels: the *S. aureus* strains Newman and USA300 JE2 with an impaired *tyrA* gene (Δ*tyrA* and Δ*tyrA* pRB473 strains) are referred as *tyrA*$^-$ and shown in purple, while the strain with native or complemented *tyrA* gene (WT and Δ*tyrA* pRB473-*tyrA* strains) are named *tyrA*$^+$ and shown in verdigris.

A, C. Growth kinetics of WT and deletion mutant strains in SNM and SNM + 100 µM tyrosine (TYR). Lines represent the mean ± SD of four biological replicates of OD_{600nm} measurements, taken every 15 minutes over 36 hours.

B, D. Barplots showing the bacterial counts of *S. aureus* cultivated on SNM agar in isolation or in the presence of the SynCom A over three days. Bars represent the mean ± SD of four biological replicates. Statistical significance was assessed using two-way ANOVA with Šídák's correction.

E. Growth kinetics of deletion and complemented mutant strains in SNM and SNM + 100 µM tyrosine (TYR). Lines represent the mean ± SD of three biological replicates of OD_{600nm} measurements, taken every 15 minutes over 26 hours.

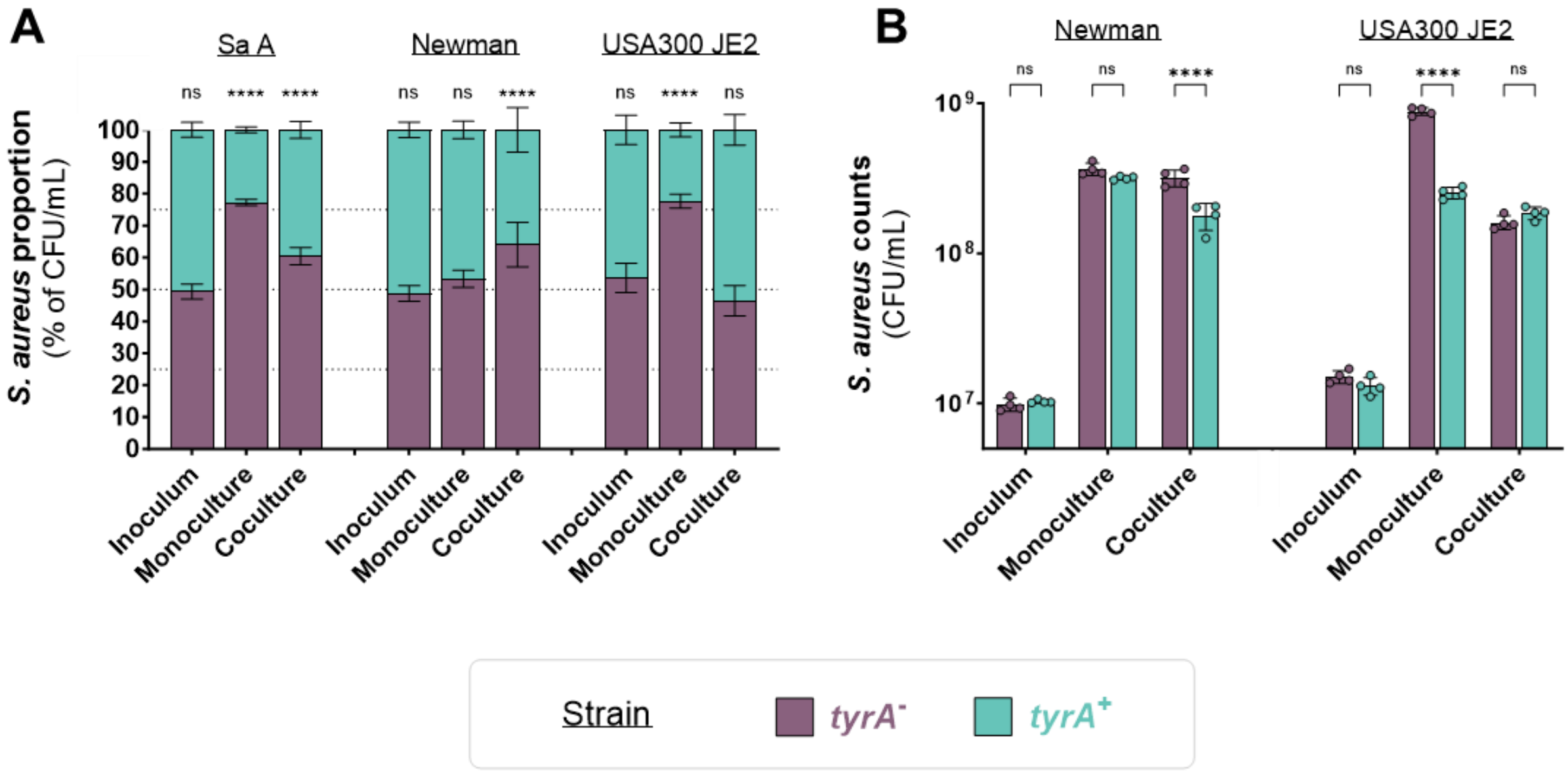

Fig. S12: Effect of *tyrA* deficiency on *S. aureus* fitness in SNM.

Barplots showing the bacterial proportion (**A**) or counts (**B**) of *tyrA*$^+$ and *tyrA*$^-$ strains of *S. aureus* in the inoculum, cultivated in SNM + tyrosine either in isolation or cocultured with each other over 24 hours. For all panels, the *S. aureus* strains with an impaired *tyrA* gene (Sa A WT, Newman and USA300 JE2 Δ*tyrA*) are referred as *tyrA*$^-$ and shown in purple, while the strains with a restored or native *tyrA* gene (Sa A-*tyrA*, Newman and USA300 JE2 WT) are named *tyrA*$^+$ and shown in verdigris. Dashed lines indicate the proportions 25%, 50% and 75% on the panel A. Bars represent the mean ± SD of four biological replicates. Statistical significance is indicated by ****P_{adj}<0.0001 (Two-way ANOVA with Šídák's correction).

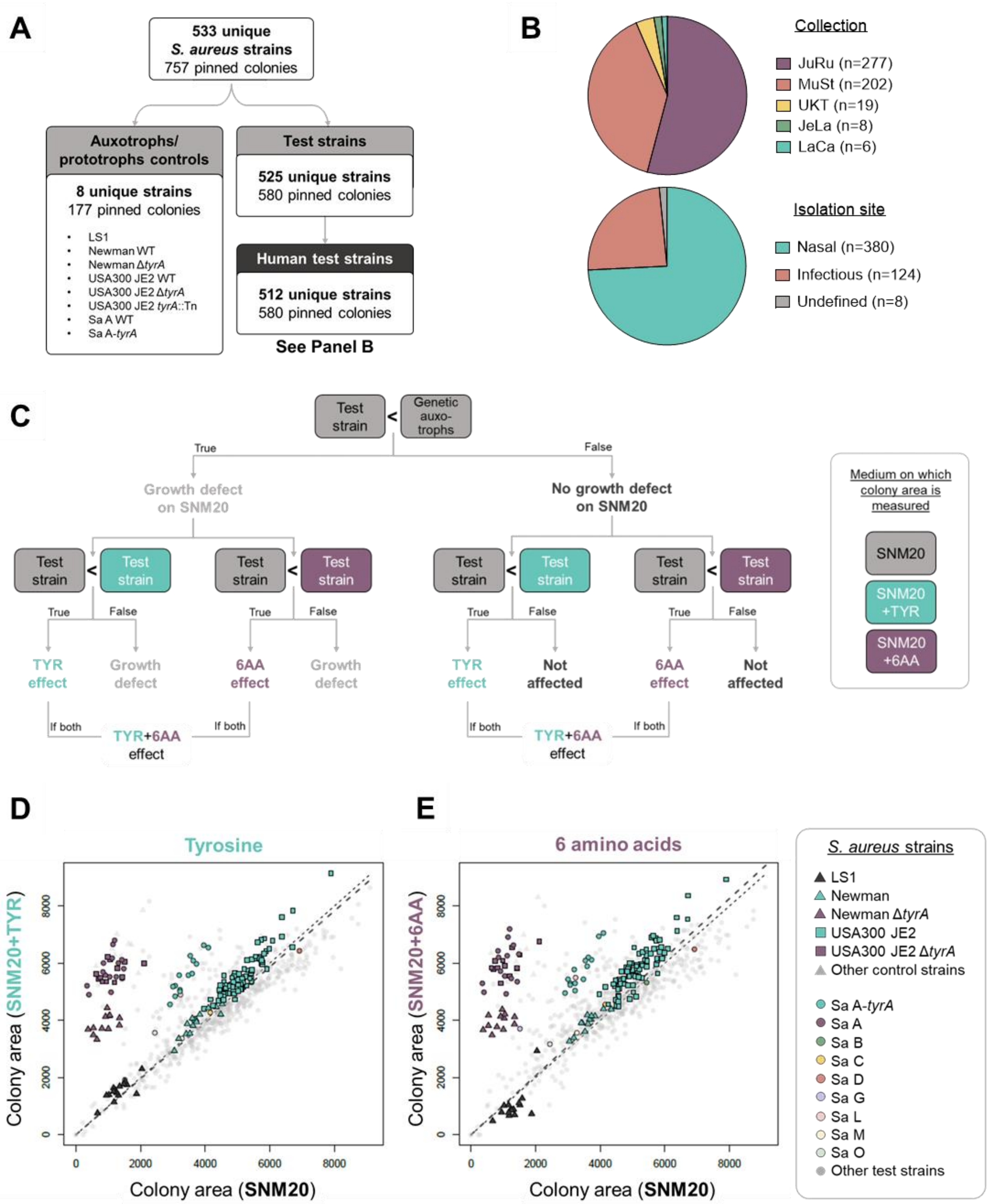

Fig. S13: Screening for phenotypic tyrosine auxotrophies in *S. aureus*.

A. Numbers of unique strains and corresponding pinned colonies screened for phenotypic auxotrophy.

B. Pie charts illustrating the collections from which the 512 human strains were obtained (top) and their isolation sites (bottom).

C. Decision tree used to analyze the growth phenotypes of *S. aureus* strains pinned on SNM20 and SNM20 supplemented with tyrosine (TYR), or the six amino acid mixture (6AA). Genetic auxotrophs were used as negative controls to identify strains with a growth defect on SNM20, and include *S. aureus* strains LS1 and *tyrA* mutants of Newman and USA300 JE2.

D, E. Dotplots showing the relationship between growth on SNM20 and SNM20 supplemented with tyrosine (**D**), or the six amino acid mixture (**E**), for the complete collection of *S. aureus* strains (n= 757 depicted colonies; n=533 unique strains). Dots represent the colony area from one biological replicate. Controls and strains of interest are shown in different shapes and colors. The diagonal dashed lines represent the linear regression corresponding to a ratio of one (thin line) or the actual linear regression (thick line).

Supplementary tables (separated Excel file)

Table S1: Complete 16S rRNA gene sequencing results.
Absolute and relative abundance of all Amplicon Sequence Variants (ASV) determined by 16S rRNA gene sequencing of nasal metagenomic DNA from eleven healthy volunteers (A-K). Each value was obtained from one replicate.

Table S2: Summary of 16S rRNA gene sequencing and strain isolation results.
Number of ASV, isolates or species identified by the two approaches for the eleven volunteers (A-K). Community State Types[10] (CST) were assigned according to the most abundant species identified by 16S rRNA gene sequencing.

Table S3: LaCa nasal strain collection.
Strains isolated from the nasal swabs of eleven healthy volunteers (A-K). For each strain, species was first identified via MALDI-MS and the abundance within the swab was determined by enumeration on agar plates. The genome of each strain was then sequenced to confirm the species identification and determine the intraspecies clonality. “LaCa identifier” designates the strain number in the collection LaCa, whereas “Special identifier” indicates the strain name used in this manuscript.

Table S4: 16S rRNA gene sequencing on SynComs mono- and co-cultures.
Relative abundance of species determined by 16S rRNA gene sequencing of frozen SynComs (Input), SynComs cultivated for 3 days on SNM agar in isolation (Mono.) or in presence of *S. aureus* (Newman, Sa A, Sa B, Sa C). R1 and R2 designate the biological replicates which were sequenced. One replicate was sequenced for the condition “Input”, and for the condition “Mono.” of SynCom K. ASVs assigned to the same species were collapsed accordingly. The “Other species” category includes all ASVs with the lowest relative abundance (<0.5%) across all samples.

Table S5: Number of BGCs and interactions identified in communities A, B, C, J, K.
BGCs and BGCs with antimicrobial potential were identified through antiSMASH analysis on the WGS data of their respective strains. Values depict the total number of BGCs, and the number of BGCs showing a medium or high similarity confidence as provided by antiSMASH.

Table S6: Amino acid relative concentrations detected by LC-MS in five SynComs.
Ratio of ^{12}C signal of the sample and ^{13}C signal of the internal standard, for 17 amino acids detected by LC-MS analysis on SynCom cultures (A, B, C, J, K). Each value was obtained from one biological replicate (four per SynCom). Optical density measurements used to normalize the data to SynCom growth are also presented.

Table S7: Non-LaCa bacterial strains used in this study.
E. coli and *S. aureus* wild-type and mutant strains, as well as *S. aureus* isolates from the collections of Munich students (MuSt), Jesper Larsen (JeLa), Justine Rudkin (JuRu) or Universitätsklinikum Tübingen (UKT). Depending on the collection, strains were identified either by MALDI-MS, isolation on *S. aureus* selective media, or whole-genome sequencing. Associated references: [11–20]

Table S8: Primers and plasmids used in this study. Associated references: [12,21]

Table S9: Culture conditions of nasal species from the LaCa collection.
Medium, oxygenation conditions and duration used for cultivating nasal species.

Table S10: Primary colony size measurements from pairwise interaction assays.
Primary colony area measurements determined using *BactoVision* for all strain pairs tested in the interaction plating experiment. Each strain was assigned an identifier from 1 to 12. When pinned, the strain x is referred to as "P_x" and when used as a background species, as "L_x". The PBS lawn control is denoted "L_0." Biological replicates are annotated "A," "B," and "C," and technical replicates (pinning repeats) are numbered from 1 to 8. "na" indicates "not attributed," when the strain pair was not tested. Missing values annotated as "nd" ("not detected") correspond to colonies that could not be reliably measured by *BactoVision* due to insufficient or no growth.

Table S11: Fold change and statistical analysis of colony size variations in pairwise interactions.
Comparison of colony sizes of each pinned strain (P_1-P_{12}) on background lawns relative to the PBS control. Fold changes were calculated as the ratio of colony area on the background lawn (L_1-L_{12}) to that on the PBS control (L_0). The adjusted *P*-values were obtained from one-way ANOVA tests comparing colony areas on PBS versus background lawns, followed by Dunnett's correction for multiple comparisons. "na" (for "not attributed") indicates when fold change and/or statistical analysis could not be performed due to missing values. "Dep" (for "dependency") denotes when a strain grew only in the presence of a background species but not on the PBS control, hindering fold-change calculation.

Supplementary references

1. van Dalen, R. *et al.* Secretory IgA impacts the microbiota density in the human nose. *Microbiome* **11**, 233 (2023).
2. F Escapa, I. *et al.* Construction of habitat-specific training sets to achieve species-level assignment in 16S rRNA gene datasets. *Microbiome* **8**, 65 (2020).
3. Kaspar, U. *et al.* The culturome of the human nose habitats reveals individual bacterial fingerprint patterns. *Environ Microbiol* **18**, 2130–2142 (2016).
4. Idelevich, E. A. *et al.* Rapid identification of microorganisms from positive blood cultures by MALDI-TOF mass spectrometry subsequent to very short-term incubation on solid medium. *Clin Microbiol Infect* **20**, 1001–1006 (2014).
5. Kolmogorov, M., Yuan, J., Lin, Y. & Pevzner, P. A. Assembly of long, error-prone reads using repeat graphs. *Nat Biotechnol* **37**, 540–546 (2019).
6. Schwengers, O. *et al.* Bakta: rapid and standardized annotation of bacterial genomes via alignment-free sequence identification. *Microb Genom* **7**, 000685 (2021).
7. Wick, R. R., Judd, L. M., Gorrie, C. L. & Holt, K. E. Unicycler: Resolving bacterial genome assemblies from short and long sequencing reads. *PLOS Computational Biology* **13**, e1005595 (2017).
8. Koren, S. *et al.* Canu: scalable and accurate long-read assembly via adaptive k-mer weighting and repeat separation. *Genome Res* **27**, 722–736 (2017).
9. Seemann, T. Prokka: rapid prokaryotic genome annotation. *Bioinformatics* **30**, 2068–2069 (2014).
10. Liu, C. M. *et al.* Staphylococcus aureus and the ecology of the nasal microbiome. *Sci Adv* **1**, e1400216 (2015).
11. Monk, I. R., Shah, I. M., Xu, M., Tan, M.-W. & Foster, T. J. Transforming the untransformable: application of direct transformation to manipulate genetically Staphylococcus aureus and Staphylococcus epidermidis. *mBio* **3**, e00277-11 (2012).
12. Monk, I. R., Tree, J. J., Howden, B. P., Stinear, T. P. & Foster, T. J. Complete Bypass of Restriction Systems for Major Staphylococcus aureus Lineages. *mBio* **6**, e00308-15 (2015).
13. Fey, P. D. *et al.* A Genetic Resource for Rapid and Comprehensive Phenotype Screening of Nonessential Staphylococcus aureus Genes. *mBio* **4**, e00537-12 (2013).
14. Duthie, E. S. & Lorenz, L. L. Staphylococcal coagulase; mode of action and antigenicity. *J Gen Microbiol* **6**, 95–107 (1952).
15. Novick, R. Properties of a cryptic high-frequency transducing phage in *Staphylococcus aureus*. *Virology* **33**, 155–166 (1967).
16. Nair, D. *et al.* Whole-genome sequencing of Staphylococcus aureus strain RN4220, a key laboratory strain used in virulence research, identifies mutations that affect not only virulence factors but also the fitness of the strain. *J Bacteriol* **193**, 2332–2335 (2011).
17. Kuroda, M. *et al.* Whole genome sequencing of meticillin-resistant *Staphylococcus aureus*. *The Lancet* **357**, 1225–1240 (2001).
18. Zhang, P., Wright, J. A., Osman, A. A. & Nair, S. P. An aroD Ochre Mutation Results in a Staphylococcus aureus Small Colony Variant That Can Undergo Phenotypic Switching via Two Alternative Mechanisms. *Front Microbiol* **8**, 1001 (2017).
19. Larsen, J. *et al.* Meticillin-resistant Staphylococcus aureus CC398 is an increasing cause of disease in people with no livestock contact in Denmark, 1999 to 2011. *Euro Surveill* **20**, (2015).
20. Young, B. C. *et al.* Severe infections emerge from commensal bacteria by adaptive evolution. *eLife* **6**, e30637 (2017).
21. Brückner, R. A series of shuttle vectors for *Bacillus subtilis* and *Escherichia coli*. *Gene* **122**, 187–192 (1992).
